# New insights into the dihydro-mureidomycin biosynthesis controlled by two unusual proteins in *Streptomyces roseosporus*

**DOI:** 10.1186/s12934-023-02260-6

**Published:** 2023-12-12

**Authors:** Ning Liu, Yang Xu, Fei Shang, Huiying Sun, Xiang Liu, Ying Huang, Huarong Tan, Jihui Zhang

**Affiliations:** 1 State Key Laboratory of Microbial Resources, Institute of Microbiology, Chinese Academy of Sciences, Beijing, 100101 China; 2 College of Life Sciences, University of Chinese Academy of Sciences, Beijing, 100049 China; 3 Analytical and Testing Center, Beijing University of Chemical Technology, Beijing, 100029 China

**Keywords:** *Streptomyces roseosporus*, Mureidomycin, Structural diversification, Natural products

## Abstract

**Background:**

Uridyl peptide compounds are renowned as a subclass of nucleoside antibiotics for their highly specific antibacterial activity against Gram-negative bacteria and the unique target of action. We previously activated the biosynthetic gene cluster of a uridyl peptide antibiotic, mureidomycin, in *Streptomyces roseosporus* NRRL 15998 by introducing an exogenous positive regulator gene *ssaA*, and the generated strain was designated as Sr-hA. This study aims to further explore mureidomycin analogs from Sr-hA as well as the collaborative roles of two wide-spread genes, *SSGG-02980* and *SSGG-03002* encoding putative nuclease/phosphatase and oxidoreductase respectively, in mureidomycin diversification.

**Results:**

In order to understand how *SSGG-02980* and *SSGG-03002* contribute to mureidomycin biosynthesis, the gene disruption mutants and complementary strains were constructed. Mass spectrometry analyses revealed that two series of pairwise mureidomycin analogs were synthesized in Sr-hA with a two-dalton difference in molecular weight for each pair. By disruption of *SSGG-03002*, only mureidomycins with lower molecular weight (MRDs, **1**–**6**) could be specifically accumulated in the mutant (∆03002-hA), whereas the other series of products with molecular weight plus 2 Da (rMRDs, **1ʹ**–**6ʹ**) became dominant in *SSGG-02980* disruption mutant (∆02980-hA). Further comprehensive NMR analyses were performed to elucidate the structures, and three MRDs (**3**, **4**, **5**) with unsaturated double bond at C5-C6 of uracil group were characterized from ∆03002-hA. In contrast, the paired rMRDs analogs (**3ʹ**,** 4ʹ**,** 5ʹ**) from ∆SSGG-02980 corresponding to **3**, **4** and** 5** were shown to contain a single bond at this position. The results verified that SSGG-03002 participates in the reduction of uracil ring, whereas SSGG-02980 antagonizes the effect of SSGG-03002, which has been rarely recognized for a phosphatase.

**Conclusions:**

Overall, this study revealed the key roles of two wide-spread families of enzymes in *Streptomyces*. Of them, oxidoreductase, SSGG-03002, is involved in dihydro-mureidomycin biosynthesis of *S. roseosporus*, whereas nuclease/phosphatase, SSGG-02980, has an adverse effect on *SSGG-03002*. This kind of unusual regulation model between nuclease/phosphatase and oxidoreductase is unprecedented, providing new insights into the biosynthesis of mureidomycins in *Streptomyces*. The findings would be of significance for structural diversification of more uridyl peptide antibiotics against Gram-negative bacteria.

**Supplementary Information:**

The online version contains supplementary material available at 10.1186/s12934-023-02260-6.

## Background

The development of high-efficiency and low-toxicity antibiotics is an imminent task due to the continuous emergence and rapid spread of resistant strains as a staggering global threat, especially the clinically intractable Gram-negative bacteria infections. The discovery of potential drug leads from microbial resources has been well accepted as a way to fight against antibiotic-resistant pathogens. With the advancement of multiple disciplinary technologies, efficient strategies are emerging for activating and rational engineering the biosynthetic pathways of natural products, including genome mining, regulatory gene manipulation, signaling modulation, metabolic flux maneuver and synthetic biology, which have accelerated the enrichment of natural products [1–3].

Nucleoside compounds are a large family of microbial secondary metabolites with broad clinical application prospects. They are generally classified into uracil-, cytosine- or adenine-derived nucleosides, and so on, such as albomycin against *Streptococcus pneumoniae*, nikkomycins and polyoxins as anti-fungal antibiotics and antiviral drug gougerotin. Uridyl peptide metabolites are promising lead compounds due to their low toxicity and specific antibacterial activity against *Pseudomonas aeruginosa*, a common pathogen resistant to various clinically used antibiotics [4]. They are expected to have no cross-resistance with currently used drugs owing to the unique target, UDP-*N*-acetylmuramic acid-pentapeptide translocase (MraY) engaging in cell wall synthesis [5]. At present, only four groups of uridyl peptide antibiotics were characterized, represented by pacidamycin from *Streptomyces coeruleorubidus* AB 1183F-64 [6], napsamycin from *Streptomyces* sp. DSM5940 [7], sansanmycin from *Streptomyces* sp. SS [8], and mureidomycin from *Streptomyces flavidovirens* [9]. They have similar core structure comprising of a 3ʹ-deoxyuridine connected with *N*-methyl-2,3-diaminobutyric acid (DABA) through 4ʹ,5ʹ-enamide bond and peptide skeleton. Taking the pacidamycin biosynthesis as an example, the common amino acids in its peptidyl group are derived from the primary metabolism, the rare amino acid DABA is produced by the conversion of threonine and ammonia, and *m*-tyrosine is converted from phenylalanine [10–12]. These amino acids are then assembled by non-ribosomal peptide synthetases (NRPS) [12, 13]. The 4ʹ,5ʹ-dehydronucleoside group is mainly synthesized from uracil through three steps of enzymatic reactions [14]. Finally, the complete structure of pacidamycin is generated following condensation [12]. It is noteworthy that the NRPs proteins responsible for peptide assembly have a highly relaxed preference of different amino acid substrates, considerably increasing the peptidyl group varieties [4]. This kind of modular assembly mode and structure features would enable the creation of new structural compounds by redesigning and modifying the biosynthetic pathways via mutational or combinatorial biosynthesis, and heterologous expression of the gene clusters. For instance, the deletion of *ssaX* gene or expression of halogenase gene *prnA* proved to be efficient in the N-terminus diversification of sansanmycin or chlorination of pacidamycin at tryptophan moiety, respectively [15, 16].

Mureidomycins are multi-component secondary metabolites, including mureidomycins A–F and the acetylated derivatives (*N*-acetylmureidomycins A–B and E–J) [17–19], with antibacterial activity against various *P. aeruginosa* strains [5]. Previously, *ssaA*, an exogenous positive regulatory gene from the sansanmycin biosynthetic gene cluster (BGC), was introduced into *Streptomyces roseosporus*, leading to the activation of mureidomycin BGC [17]. However, since the product profile was extremely complicated, numerous mureidomycin components produced in the strain could not be isolated and characterized explicitly. Here, we further exploit this valuable resource via mutasynthesis, and characterize key genes involved in the uracil reduction, which would be beneficial for the structural diversification of uridyl peptide antibiotics.

## Results

### Bioinformatics analyses of *SSGG-02980* and *SSGG-03002* genes in a mureidomycin BGC

In our previous study, a cryptic mureidomycin biosynthetic gene cluster (*mrd* BGC) was characterized in *S. roseosporus* NRRL 15998 and activated by introducing *ssaA*, an exogenous positive regulatory gene [17]. The resultant recombinant strain referred as Sr-hA in the present work was used for further exploiting the potential of mureidomycin diversification through mutagenesis of key genes.

**Fig. 1:**
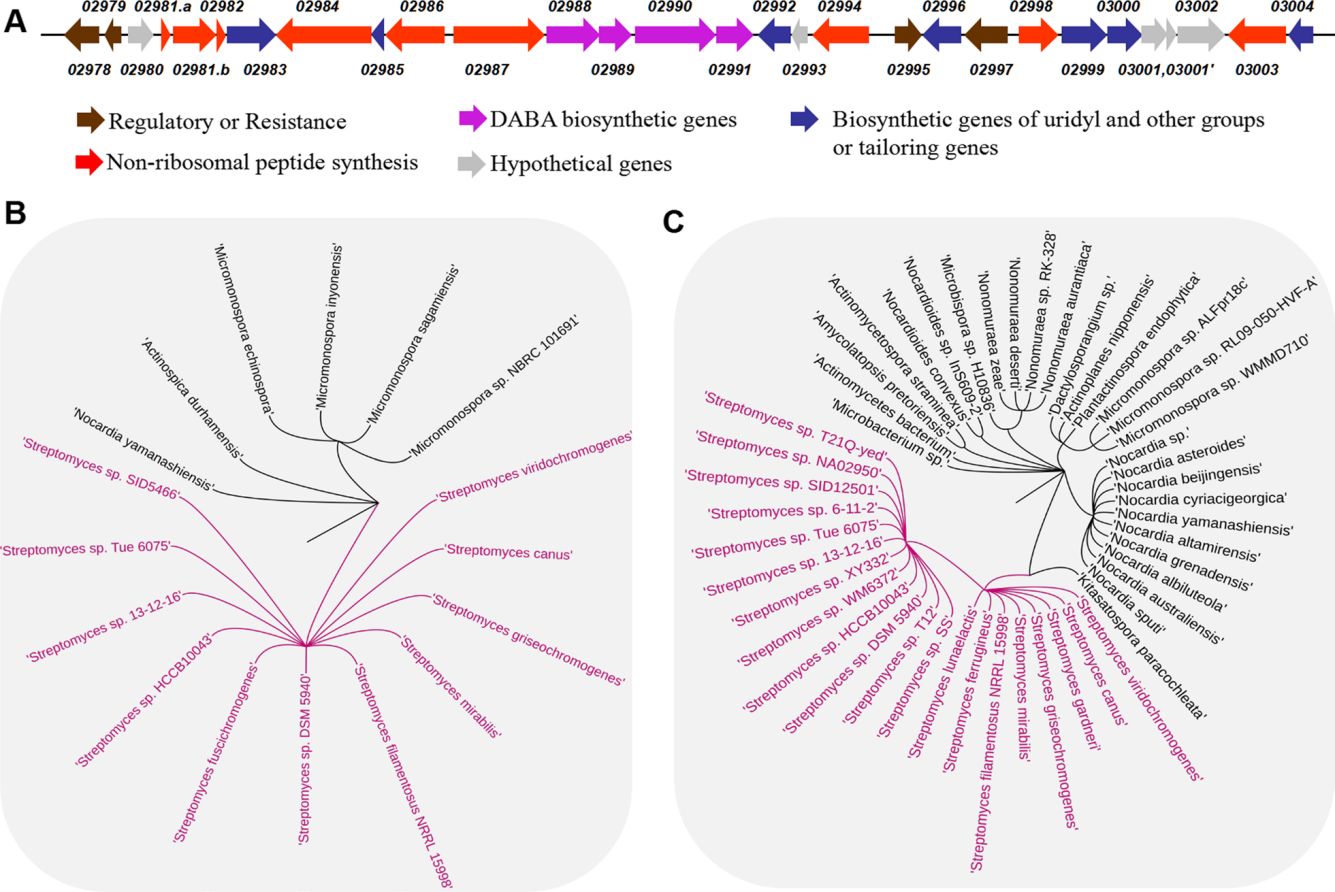
Distribution of SSGG-03002 and SSGG-02980 homologous proteins in actinomycetes. **A** Organization of mureidomycin biosynthetic gene cluster. **B**, **C** The distribution of SSGG-03002 and SSGG-02980 homologous proteins in actinomycetes, respectively. *Streptomyces* species are indicated by purple fonts

*SSGG-02980* and *SSGG-03002* in *mrd* BGC are hypothetical genes (Fig. 1A). Blast analyses of the encoded proteins SSGG-03002 (Accession No. EFE75635.1 in GenBank) and SSGG-02980 (Accession No. EFE75613.1 in GenBank) against NCBI database revealed that they share low or moderate identity with putative F420/FMN-dependent oxidoreductase and phosphatase, respectively (Additional files 1, 2). Most of these proteins exist in prokaryotes, and a small number of them can be found in eukaryotes. Specifically, 42 entries of SSGG-03002 homologous proteins were found, and 19 of them are distributed in 11 *Streptomyces* species (Fig. 1B). Likewise, 201 entries of SSGG-02980 homologous proteins were found, 31 out of which are from 20 *Streptomyces* species (Fig. 1C). Further antiSMASH analyses indicated that, among the actinomycetes hits of SSGG-03002 homologous proteins, 53% of them contain both *SSGG-03002* and *SSGG-02980* coexisting in the same BGCs, 10% contain both genes but *SSGG-03002* is out of the identified BGCs, while the remaining strains contain only discrete *SSGG-03002*. For *SSGG-02980* homologs in *Streptomyces,* 57% of them appear as stand-alone genes instead of coexisting with *SSGG_03002*, and 75% situate within in BGCs*.* So, *SSGG-03002* is present along with *SSGG-02980* as a pair in most cases, while *SSGG-02980* might be more independent. To date, there are no reports available about the exact activities of these proteins in *Streptomyces* apart from partial domain sequence alignment. Considering the universality and wide distribution, SSGG-03002 and SSGG-02980 are expected to have important roles in *Streptomyces* yet to be resolved. Thus, the elucidation of their functions would be of great necessity.

### The effects of *SSGG-02980* and *SSGG-03002* disruption on mureidomycin biosynthesis in *S. roseosporus*

**Fig. 2:**
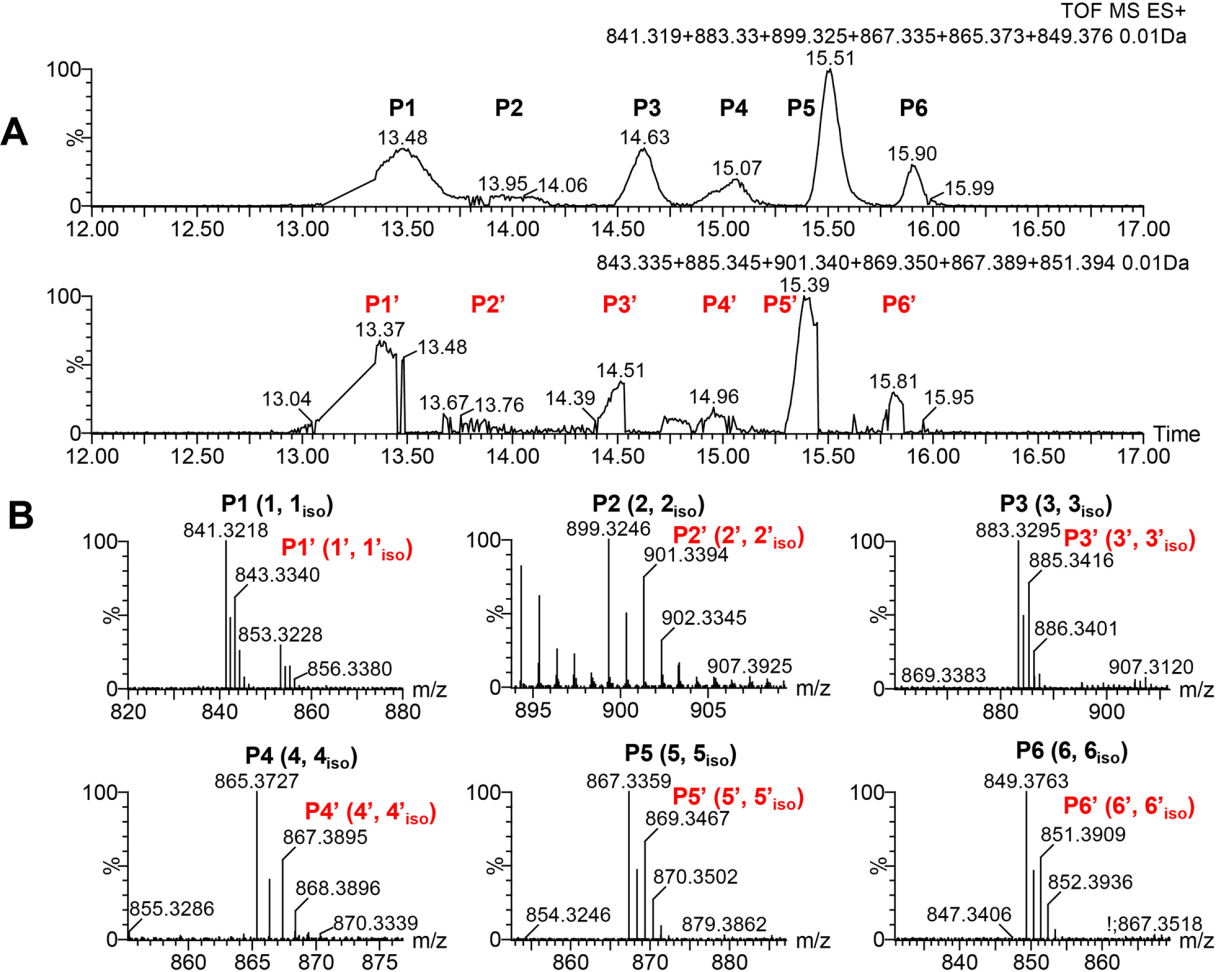
UPLC-HRMS analyses of mureidomycins in strain Sr-hA. **A** Extracted ion currents of mureidomycins in Sr-hA (P1-P6 and their corresponding peaks with two units increase in *m/z* of [M+H]^+^, P1ʹ–P6ʹ). **B** Mass spectra of the extracted quasi-molecular ions of mureidomycins. The minor content isomers of the corresponding dominant compounds are indicated by ‘iso’ footnote

To understand whether and how *SSGG-02980* and *SSGG-03002* contribute to mureidomycin biosynthesis and derivatives generation, the disruption mutant of *SSGG-02980* (∆02980-hA) and its complementary strain (∆02980c-hA) were constructed and verified by PCR (Additional file 3: Fig. S1). Likewise, the mutant of *SSGG-03002* (∆03002-hA) and complementary strain ∆03002c-hA were obtained (Additional file 3: Fig. S1). All the strains, plasmids and primers used in this work are provided separately (Additional file 3: Tables S1-S3). The strains were then evaluated for the production of mureidomycins. UPLC‒HRMS analyses revealed that the starting strain Sr-hA produced multiple peaks (P1–P6, P1ʹ–P6ʹ) overlapped with each other with retention times spanning from 12 to 17 min (Fig. 2A, Table 1). P1–P6 and P1ʹ–P6ʹ were indicated to be pairwise, and the molecular weight of the compounds contained in P1–P6 (MRDs) is 2 Da lower than their corresponding analogs in P1ʹ–P6ʹ (rMRDs) (Fig. 2B). Additionally, the retention time of rMRDs on UPLC was shifted to approximately 0.1 min earlier than that of the paired MRD peaks (Fig. 2A). Further analyses of the fermentation products in the gene disruption mutants showed that, in ∆03002-hA, rMRDs were abolished and only MRDs were retained; whereas rMRDs were accumulated in ∆02980-hA with a significant decrease of MRDs (Additional file 3: Fig. S2 and S3). Moreover, the production of most of these compounds in the complementary strain, ∆02980c-hA, returned to a similar level as that in Sr-hA (Additional file 3: Fig. S4). Intriguingly, in situ complementation of *SSGG-03002* in ∆03002-hA (∆03002c-hA) restored the biosynthesis of rMRDs, but not in the ex situ complementation, suggesting that some kind of location effects might exist for SSGG-03002 to function properly (Additional file 3: Fig. S5). Finally, the double disruption mutant (∆02980/03002-hA) was constructed and verified by PCR (Additional file 3: Fig. S1). In ∆02980/03002-hA, only MRDs could be detected, similar to the phenotype of ∆03002-hA (Additional file 3: Fig. S6). These results verified that *SSGG-02980* and *SSGG-03002* participated in the switch between MRD and rMRD, but the precise difference on the structures remained to be characterized. For clarity, the peaks of P1–P6 and P1ʹ–P6ʹ, *m/z* of [M+H]^+^, the dominant component and its corresponding isomer as minor content in each peak (see below for more details) are coded and summarized in Table 1.

**Table 1:** The putative paired quasi-molecular ions and their codes

Peaks	[M+H]^+^ Measured/Calculated	Dominant compounds	Minor isomers	Peaks	[M+H]^+^ Measured/Calculated	Dominant compounds	Minor isomers
P1	841.3218/ 841.3190	**1**	**1**_**iso**_	P1	843.3340/ 843.3339	**1**	**1**_**iso**_
P2	899.3246/ 899.3245	**2**	**2**_**iso**_	P2	901.3394/ 901.3402	**2**	**2**_**iso**_
P3	883.3295/ 883.3296	**3**	**3**_**iso**_	P3	885.3416/ 885.3453	**3**	**3**_**iso**_
P4	865.3727/ 865.3732	**4**	**4**_**iso**_	P4	867.3895/ 867.3888	**4**	**4**_**iso**_
P5	867.3359/ 867.3347	**5**	**5**_**iso**_	P5	869.3467/ 869.3503	**5**	**5**_**iso**_
P6	849.3763/ 849.3783	**6**	**6**_**iso**_	P6	851.3909/ 851.3939	**6**	**6**_**iso**_

### Fermentation and isolation of MRDs and rMRDs

To determine the structures of mureidomycin analogs as mentioned above (MRDs and rMRDs), large-scale fermentation of strains ∆02980-hA and ∆03002-hA and preparative HPLC purification were undertaken. The [M+H]^+^ values of peaks P1, 1ʹ, 2, 6, and 6ʹ (*m/z* 841.3218, 843.3340, 899.3246, 849.3763, 851.3909) are identical to those of previously reported mureidomycin A [17, 18], mureidomycin B [17, 18], *N-*acetylmureidomycin E [19], *N-*acetylmureidomycin I and J (Fig. 2B) [17], respectively. Hence, we focused on other abundant peaks, P3 (4.1 mg), P4 (3.8 mg) and P5 (2.9 mg), from the fermentation broth of ∆03002-hA, as well as P3ʹ (3.8 mg), P4’ (2.1 mg) and P5ʹ (0.7 mg) from ∆02980-hA. All of them were obtained as light yellow or transparent solids and subjected to NMR analyses. Moreover, in each peak of P3, 4, 5, P3ʹ and P4ʹ, a dominant component and minor isomer were indicated based on NMR data, so the structural elucidation was primarily carried out with the more abundant compound in each peak (Fig. 2B, Table 1).

### Structural elucidation of the representative MRDs compounds

In P3, the molecular formula of the dominant component (**3)** was established to be C_40_H_50_N_8_O_13_S ([M+H]^+^ measured as *m/z* 883.3295, calculated 883.3296) through high-resolution mass spectrometry (HRMS) (Fig. 2B), indicating 20 unsaturation degrees in the structure. Three methyl groups (1.87, 2.03 and 3.03 ppm) appeared as singlets, and a methyl group appeared as a doublet (1.19 ppm) in the ^1^H-NMR spectrum (Additional file 3: Figs. S7–S12, Table S4). According to the ^13^C-NMR, Dept135 and HSQC spectra, carbon signals were present as follows: ten carbonyl or phenolic carbons (152.1, 158.4, 158.6, 159.7, 165.9, 168.3, 173.1, 174.2, 175.0 and 176.4 ppm) between 150–180 ppm, 12 olefinic or aromatic carbons (114.7, 114.9, 117.0, 117.4, 121.3, 121.9, 130.4, 130.7, 139.8, 140.1, 141.2, and 143.7 ppm) between 110–150 ppm, six C–O or C–N carbons (52.5, 52.5, 54.3, 55.9, 56.8 and 73.9 ppm) between 50–80 ppm, nine other alkyl carbons (14.2, 15.4, 22.3, 31.1, 31.1, 33.5, 34.9, 38.3 and 39.1 ppm) between 10–45 ppm, and three possible olefinic, di-oxygenated or imine carbons (94.5, 98.3 and 103.5 ppm) (Additional file 3: Table S4).

**Fig. 3:**
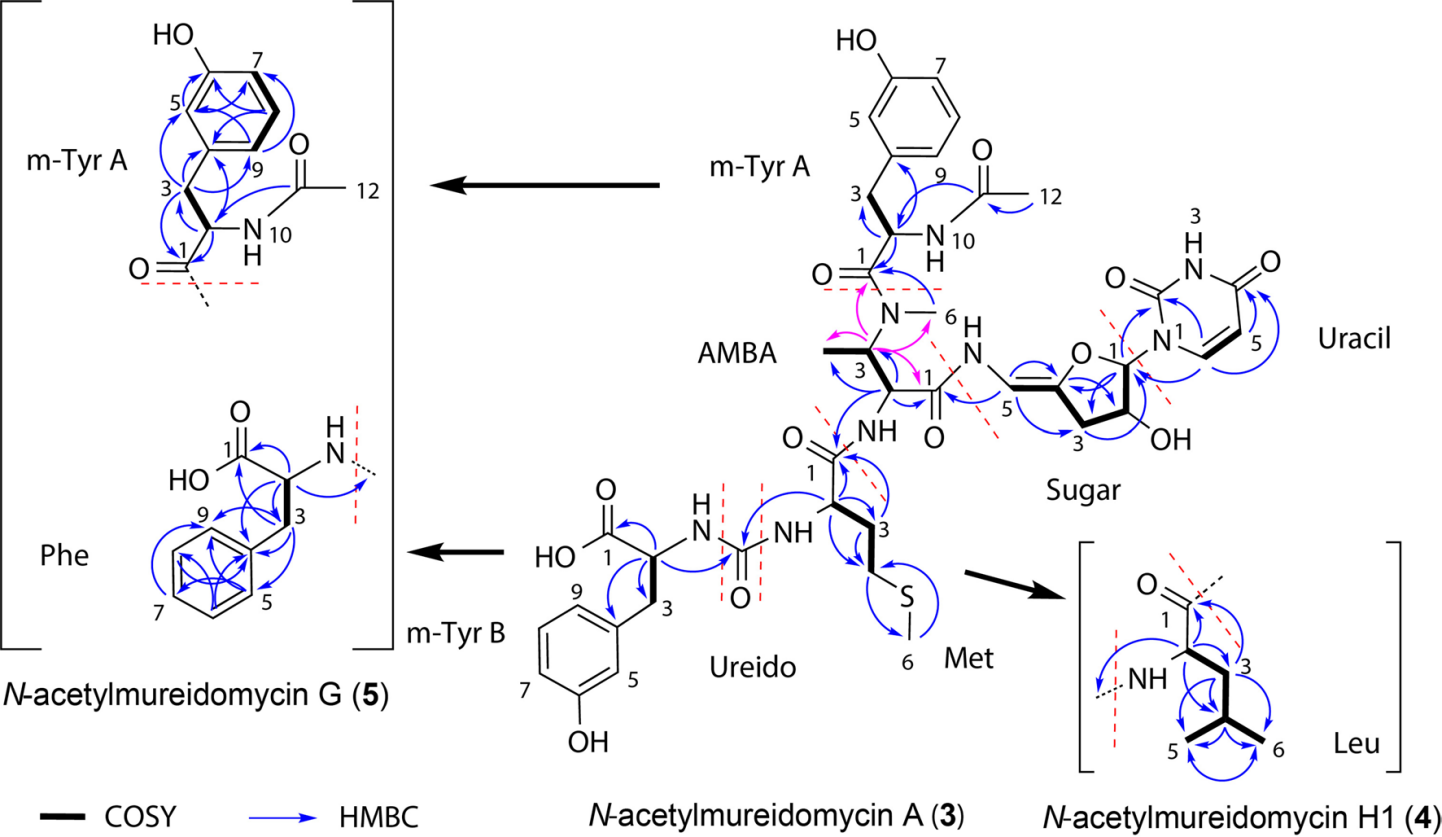
Long-range ^1^H, ^13^C-couplings in H–H COSY and HMBC of compounds **3**–**5**

Further elucidation of the uridine and 2-amino-3-methylaminobutyric acid (AMBA) structure of **3** was carried out based on the interpretation of 1D/2D-NMR data and comparison of the ^13^C-NMR with that of *N-*acetylmureidomycin E (Fig. 3) [17]. Unbroken COSY correlations established the connectivity from Uracil-H5 to Uracil-H6, Sugar-H1 to Sugar-H5, AMBA-H2 to AMBA-H4, Met-H2 to Met-H4, and *m*-tyrosine A (*m*-Tyr-A)-H2 to *m*-Tyr-A-H3. HMBC correlations from Uracil-H5 to Uracil-C4 and Uracil-H6 to Uracil-C2 and C4, combined with the comparison of ^13^C-chemical shifts of Uracil-C2, C4, C5 and C6 with those of *N-*acetylmureidomycin E, suggested a uracil-ring system. The HMBC correlation from Sugar-H1 to Sugar-C4, combined with the COSY correlation from Sugar-H1 to Sugar-H5, established a sugar ring. Furthermore, the ^13^C-chemical shifts of Sugar-C4 (143.7 ppm) and C5 (98.3 ppm) indicated that the connection between them is a double bond, where the signal of C5 was shifted to a higher field as compared to that of a regular double bond due to the attachment of an amino group. HMBC correlations from AMBA-H2 to AMBA-C1, C3 and C4 established the core structure of AMBA. Although neither the HMBC signal of N-methyl group at AMBA-H6 position to AMBA-C3 nor that of AMBA-H3 to AMBA-C6 could be observed due to spatial distance, the correlation of AMBA-H3 with AMBA-C6 in the lower content isomer **(3**_**iso**_**)** of **3** illustrated the integrity of AMBA core structure (purple arrow in Fig. 3).

Next, the peptidyl fragment of **3** was determined. The HMBC correlations from Met-H2 and H3 to Met-C1 and C4, as well as H6 to C4, established a methionine-type core (Met), where the chemical shifts of Met-C4 (31.1 ppm) and C6 (15.4 ppm) confirmed that they are bridged by sulfur atoms rather than oxygen or nitrogen atoms. Signals of C1 to C4 in *m*-tyrosine A and B (*m*-Tyr-A and *m*-Tyr-B) could be assigned by HMBC. The assignments of C5 to C9 in *m*-Tyr-A and *m*-Tyr-B were mainly based on the comparison of NMR spectra with those of *N-*acetylmureidomycin G [17], in which *m*-Tyr-B was substituted with phenylalanine, so precise assignment of signals for *m*-Tyr-A was achieved.

Finally, the HMBC correlations from Uracil-H6 to Sugar-C1, Sugar-H5 to AMBA-C1, AMBA-H2 to Met-C1, Met-H2 and *m*-Tyr-A-H2 to Ureido of **3** (purple arrow in Fig. 3; Additional file 3: Table S4), as well as AMBA-H3 to m-Tyr-A-C1 in its isomer assembled all the moieties (Uracil, Sugar, AMBA, Met, Ureido, *m*-Tyr-A and B) of **3,** and its structure was consequently determined to be *N*-acetylmureidomycin A [17]. In addition, precise assignment of ^1^H- and ^13^C-NMR data revealed that the trivial differences in chemical shifts of **3** and **3**_**iso**_ were present at moieties Uracil-6, AMBA-3, AMBA-6 and m-Tyr-A3, suggesting that **3**_**iso**_ might be an isomer of **3** named iso-*N*-acetylmureidomycin A (Additional file 3: Table S4).

The molecular formulae of the dominant components in P4 and P5 were established through HRMS to be C_41_H_52_N_8_O_13_ (**4,** [M+H]^+^ measured as *m/z* 865.3727, calculated 865.3732) and C_40_H_50_N_8_O_12_S (**5**, [M+H]^+^ measured as *m/z* 867.3359, calculated 867.3347) (Fig. 2B), respectively. So **4** should be the desulfur product of **3** with one more methine incorporated, and **5** is the deoxygenated product of **3**. Further comparison of the mass spectra indicated that the major component **4** in P4 was similar to the previously reported *N*-acetylmureidomycin H [17], but the fragment between Ureido and AMBA was leucine (Leu) rather than Ile proposed in *N*-acetylmureidomycin H by NMR analysis. This conclusion was supported by the COSY correlation from Leu-H2 to Leu-H6 and HMBC signals from Leu-H3 to Leu-C1, C2, C4, C5, and C6 (Fig. 3, Additional file 3: Figs. S13–S18). Therefore, we assigned it a new name, *N*-acetylmureidomycin H1 (Additional file 3: Table S5). Similarly, the major component in P5 was determined to *N*-acetylmureidomycin G (**5**) [17], and the main difference from **3** is that *m*-Tyr B fragment in **3** is replaced with phenylalanine (Phe). This deduction was supported by the chemical shifts of Phe-H6 (7.23 ppm) and Phe-C6 (129.4 ppm), as well as multiple HMBC signals among the hydrogen and carbon atoms of Phe (Fig. 3, Additional file 3: Figs. S19–S24, Table S6).

### Structural elucidation of the representative rMRDs

The structures of rMRDs were determined mainly based on the comparisons of mass spectra and NMR data with those of MRDs. HRMS revealed the molecular formulae of rMRDs in peaks P3’-5’ to be C_40_H_52_N_8_O_13_S ([M+H]^+^ measured as *m/z* 885.3416, calculated 885.3453), C_41_H_54_N_8_O_13_ ([M+H]^+^ measured as 867.3895, calculated 867.3888) and C_40_H_52_N_8_O_12_S ([M+H]^+^ measured as *m/z* 869.3467, calculated 869.3503) (Fig. 2B), respectively, indicating two more hydrogen atoms are contained in rMRDs than their corresponding paired peaks MRDs, which was further supported by NMR data. For example, most of the ^1^H- and ^13^C-NMR data of **3ʹ** exhibited high identity with those of component **3** (*N*-acetylmureidomycin A), illustrating the similar skeletons were contained. The only differences exist in the ^1^H-/^13^C-chemical shifts of Uracil-5 and Uracil-6, which are 5.59/103.5 ppm and 7.12/141.2 ppm in *N*-acetylmureidomycin A, supporting a double bond, while a single bond was indicated by the signals 2.57–2.65/31.8 ppm and 3.08/37.7 ppm in **3ʹ**. Further COSY and HMBC correlations confirmed this deduction (Additional file 3: Figs. S25–S30). Thus, **3ʹ** should be *N*-acetylmureidomycin B as previously described (Additional file 3: Table S7) [17]. Similarly, **4ʹ** was determined to be an analog of *N*-acetylmureidomycin H1 with a saturated single bond between Uracil-C5 and Uracil-C6 based on NMR analyses, a novel compound named *N*-acetylmureidomycin H2 (Additional file 3: Figs. S31–36, Table S8).

**Fig. 4:**
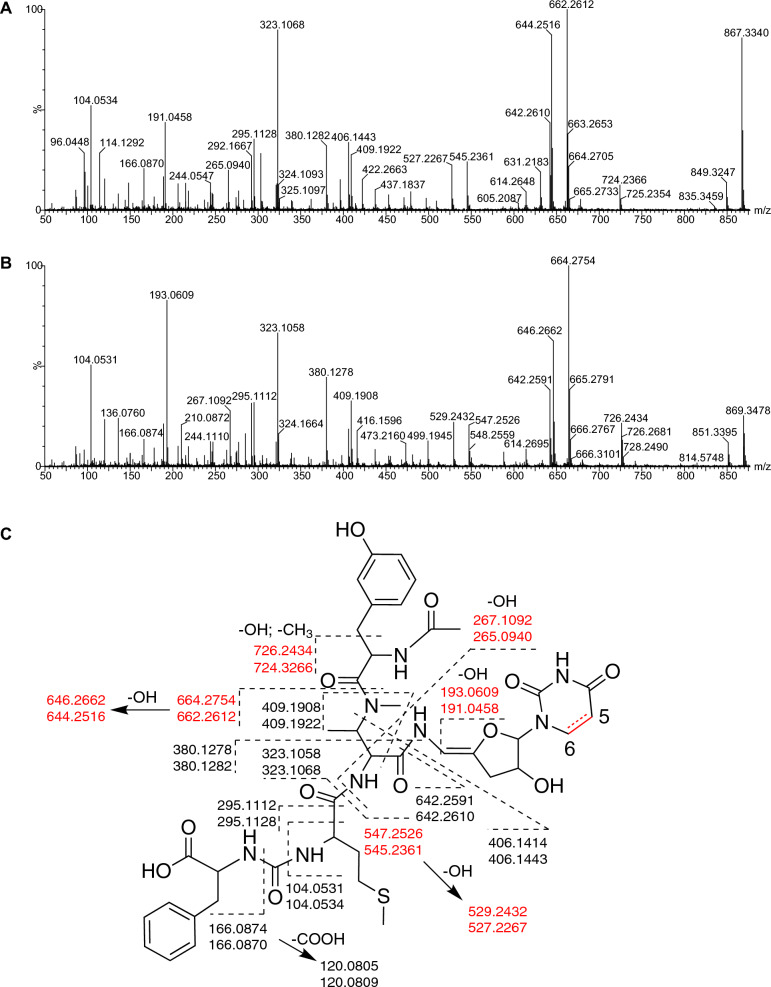
MS/MS analyses of **5ʹ** and **5**. **A**, **B**, MS/MS spectra of compounds **5** and **5ʹ**, respectively. **C**, MS/MS interpretations of **5ʹ** and **5**. Each structural fragment is labeled with two *m/z* values based on the mass spectra of **5** and **5ʹ**, respectively. Differentiated MS data are highlighted with red color

Owing to the low yield, we only collected HR-MS/MS (Fig. 4A, B), ^1^H- and ^13^C-NMR data of **5ʹ** from the *SSGG-02980* mutant strain, which contains two more hydrogen atoms than **5** (Additional file 3: Figs. S37, 38). A fragment of *m/z* 193.0609 in **5ʹ** was comparable to *m/z* 191.0458 of **5,** further confirming that the two extra hydrogen atoms are situated in the sugar-uracil moiety of **5ʹ** (Fig. 4C). In addition, based on the chemical shifts at 97.1, 143.1, 155.2, and 172.5 ppm in the ^13^C-NMR of **5ʹ** (Additional file 3: Figs. S37, S38), double bonds at Uracil-C2 to C4, and Sugar-C4 to C5 were assigned. Therefore, the saturated bond was deduced to be at Uracil-C5 and C6. Compound **5ʹ** was finally characterized as *N*-acetylmureidomycin F [17], the paired compound of **5**.

The pairwise quasi-molecular ions of [M+H]^+^ *m/z* 899.3246 and 901.3394 (P2 and P2ʹ) were detected in the HRMS profile of Sr-hA. Due to insufficient amount of P2ʹ, no NMR data were collected. The former corresponds to our previously reported *N*-acetylmureidomycin E [17], so the latter might be the reduced form of *N*-acetylmureidomycin E at uracil C5-C6 bond. It was preliminarily assigned a new name *N*-acetylmureidomycin K (Additional file 3: Fig. S39) [17].

**Fig. 5:**
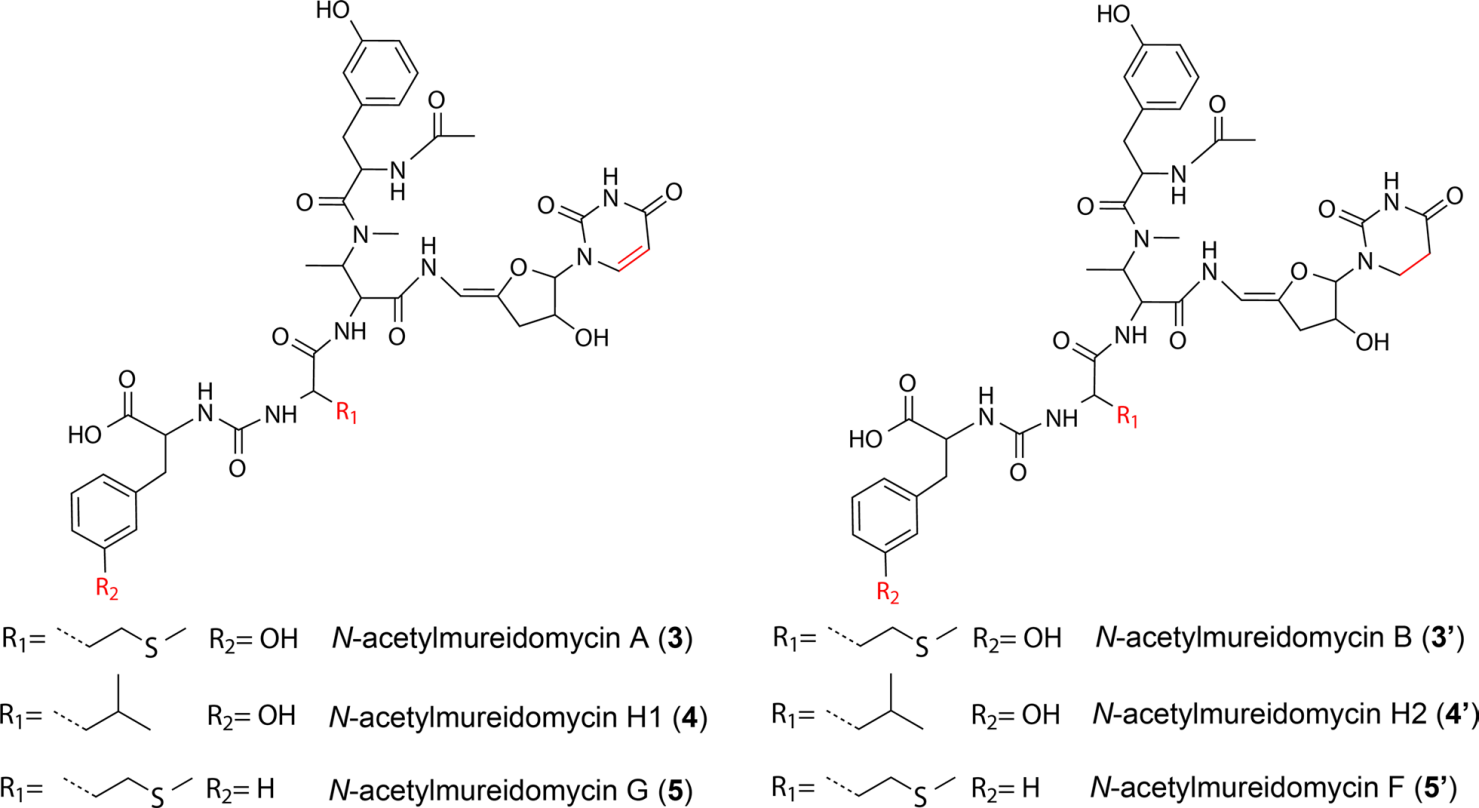
The structures of mureidomycin analogs MRDs (**3**-**5**) and rMRDs (**3ʹ**-**5ʹ**)

Together, the MRDs and rMRDs identified in this study are summarized in Table 2, and their structures are shown in Fig. 5. In addition, five *N*-acetylmureidomycin isomers were also indicated.

**Table 2:** Summary of the characterized mureidomycin analogs from *S. roseosporus*

Code	name	Isomers	Code	name	Isomers
**1**	Mureidomycin A	NA	**1ʹ**	Mureidomycin B	NA
**2**	*N-*acetyl Mureidomycin E	NA	**2ʹ**	*N-acetyl Mureidomycin K	NA
**3**	*N-*acetyl Mureidomycin A	*iso-N-acetyl Mureidomycin A	**3ʹ**	*N-*acetyl Mureidomycin B	*iso-*N*-acetyl Mureidomycin B
**4**	*N-acetyl Mureidomycin H1	*iso-N-acetyl Mureidomycin H1	**4ʹ**	*N-acetyl Mureidomycin H2	*iso-N-acetyl Mureidomycin H2
**5**	*N-*acetyl Mureidomycin G	*iso-*N*-acetyl Mureidomycin G	**5ʹ**	*N-*acetyl Mureidomycin F	NA
**6**	*N-*acetyl Mureidomycin I	NA	**6ʹ**	*N-*acetyl Mureidomycin J	NA

*NA* not available

^*^Firstly identified compounds in this study

### The effects of *SSGG-2980* disruption on the expression of *SSGG-03002*

Since SSGG-02980 and -03002 are involved in the control of dihydro-mureidomycin biosynthesis, we are interested to understand the intrinsic relationship between them. According to bioinformatic analyses, SSGG-02980 was predicted to be a phosphatase family protein, so we carried out some investigations about the effect of SSGG-02980 on SSGG-03002.

First of all, the transcription of *SSGG-03002* was analyzed by RT-qPCR (Additional file 4: Fig. S40). The elevated transcription of *SSGG-03002* was detected in *SSGG-02980* disruption mutant (∆02980-hA), suggesting that SSGG-02980 could have repressive roles on the transcription of *SSGG-03002*; meanwhile, rather unexpectedly, the complementation of *SSGG-02980* could not reverse the transcription level of *SSGG-03002* to that in the starting strain Sr-hA, but somehow further increased it. This was inconsistent with the product profile of the corresponding strain (∆02980c-hA), in which the MRDs yield was restored to the similar level as that in Sr-hA by *SSGG-02980* complementation. We speculated that SSGG-02980 might function on SSGG-03002 via multiple routes in addition to transcriptional regulation. More studies are needed to elucidate the mechanisms thoroughly.

On the other hand, the possible post-translational modification of SSGG-02980 on SSGG-03002 protein was analyzed. Because SSGG-02980 protein tended to precipitate quickly during isolation for its poor solubility, we applied the in vivo approach to determine the phosphorylation changes of SSGG-03002. Two expression systems of *SSGG-03002* with or without the co-expression of *SSGG-02980* in *E. coli* were constructed, from which the recombinant proteins were purified to homogeneity (Additional file 4: Fig. S41) and subjected to mass spectrometry analysis (Additional file 4: Method S1). The results showed that the reproducible phosphorylation sites on recombinant SSGG-03002 could be at threonine (T304), serine (S302) and tyrosin (Y305), so we focused on the changes of these amino acid residues. By comparison of the sequencing data, we did not find significant differences in the phosphorylation level of recombinant SSGG-03002 proteins expressed with/without SSGG-02980 (Additional file 4: Fig. S42)*,* suggesting that SSGG-02980 might not affect the activity of SSGG-03002 at post-translational level for the *E. coli*-derived recombinant proteins. So the correlation between SSGG-02980 and SSGG-03002 was complicate, and the exact interactions remained to be illustrated.

### The proposed mode of SSGG-02980 and SSGG-03002 controlling dihydro-mureidomycin biosynthesis

**Fig. 6:**
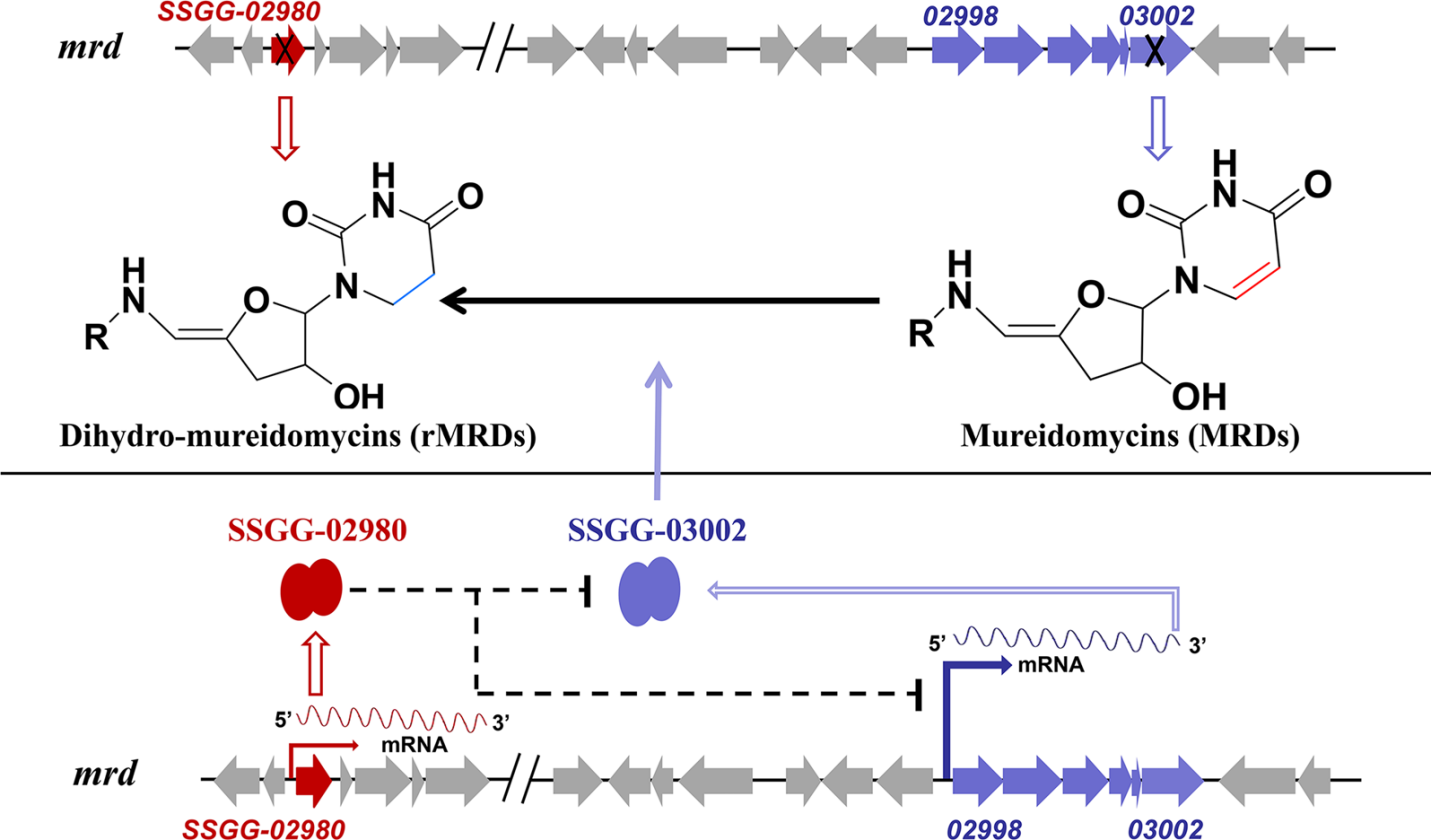
The proposed regulation of SSGG-02980/03002 on mureidomycin biosynthesis

Based on the results described above, the correlation between SSGG-02980 and SSGG-03002, and their roles in mureidomycin biosynthesis were proposed (Fig. 6). We demonstrated that disruption of *SSGG-03002* led to the accumulation of mureidomycins with unsaturated C5–C6 bond in uracil ring (MRDs), while *SSGG-02980* mutant primarily produced the dihydro-mureidomycins (rMRDs). Along with the bioinformatics data, SSGG-03002 was proven to engage in the reduction of uracil ring, while SSGG-02980 had adverse effects. Based on the potential phosphatase activity of SSGG-02980, we hypothesized that SSGG-02980 might disable SSGG-03002 function directly or indirectly via down-regulating the gene transcription or translation of the protein, reducing the enzyme activity and stability, and so on, but the precise mechanisms remain to be elucidated. Nevertheless, both enzymes collaboratively control dihydro-mureidomycin biosynthesis, representing a unique biosynthetic pathway ‘switch’ of uridyl peptide antibiotics, and the present findings would facilitate the enrichment of uridyl peptide antibiotics.

## Discussion

There is an urgent need to cope with the antibiotic crisis and resistant pathogens, especially those with cross-resistance to multiple drugs. Mining of genomes with multi-omics techniques and rational modification of compounds are quick approaches for expanding the capacity of bioactive products libraries [20]. Uridyl peptide compounds are emerging as a new family of drug leads for their unique activities specifically against the Gram-negative bacteria *Pseudomonas*; hence, they hold great potential in clinical applications. Additionally, the modular assembly feature of the uridyl and peptidyl groups endowed them with superior flexibility in creating diverse structures. In this study, two essential enzymes, SSGG-02980 and SSGG-03002, responsible for the reduction of uracil ring in mureidomycin were determined, and two series of mureidomycin analogs were characterized, demonstrating the feasibility of mutational biosynthesis in compound diversification.

Uridyl peptide antibiotics are usually synthesized as an array of multiple components, providing rich structures of metabolites but also with inconvenience in the isolation of interesting products. Disruption or overexpression of some key biosynthetic genes is a way of generating novel structural metabolites or simplifying the product constituents to favor the isolation of target molecules. In our previous work, a cryptic BGC of mureidomycins was activated by overexpression of the exogenous regulatory gene *ssaA* in *S. roseosporus* NRRL15998. We preliminarily identified the chemical structures of some mureidomycins, and clarified the activation mechanism of *mrd* gene cluster [17, 21]. However, due to the low abundance and complexity of the product profiles, the structural elucidation was mainly based on mass spectral analyses except for *N*-acetylmureidomycins E and A. Here, since two series of mureidomycins containing the oxidized or reduced forms of uracil were accumulated in ∆03002-hA and ∆02980-hA respectively, the product profile of each mutant was significantly simplified. As a result, compounds **3–5** and **3ʹ–5ʹ** were purified, and extensive NMR analyses were performed to determine their structures explicitly, including two novel mureidomycin analogs. Meanwhile, five isomers with lower abundance were indicated on the NMR spectra. Hence, characterization of the two unrecognized SSGG-02980 and SSGG-03002 provided an effective approach for generating uridyl peptide analogs via mutational biosynthesis.

As illustrated in the biosynthetic pathways, uridine group in the structure of uridyl peptide compounds is derived from uracil, and the reduction of C5–C6 double-bond was supposed to be catalyzed by some specific enzymes. A homologous protein of SSGG-03002*,* NpsU (accession number ADY76683.1 in GenBank), was identified through BLAST analysis, which shared 94.19% identity with SSGG-03002 (Additional file 4: Fig. S43). Previously, NpsU was shown to be involved in the biosynthesis of dihydrouracil napsamycins based on low-resolution MS analysis [7], yet it was necessary to further clarify which double-bond was reduced in these compounds. In the present study, both genetic approach and NMR analyses unambiguously demonstrated that SSGG-03002*,* a putative F420/FMN-dependent oxidoreductase, was responsible for reducing the double-bond at C5–C6 of the uracil moiety in mureidomycins. More interestingly, a putative phosphatase of *Streptomyces*, SSGG-02980, was found to possess antagonistic effect on SSGG-03002. How SSGG-02980 exerts its effect on SSGG-03002 remains elusive at the moment apart from the preliminary transcriptional inhibition on *SSGG-03002*. Also, the assumption of protein de-phosphorylation by SSGG-02980 was not evidenced for the *E. coli-*derived recombinant SSGG-03002 based on protein sequencing. So, other possibilities could be speculated, such as modulations on SSGG-03002 enzymatic activity, protein expression or the stability. Further elucidation of the exact regulation mechanisms requires substantial optimizations on in vitro activity evaluation approaches. Together, this kind of relationship between SSGG-02980 and SSGG-03002 was completely unrecognized previously in *Streptomyces*. The findings would be significant for understanding the roles of these two families of proteins in other species since they spread fairly wide in microorganisms.

Based on bioinformatics analyses, an F420 or FMN-binding domain was predicted in SSGG-03002. We attempted to set up enzymatic assays of SSGG-03002 to determine its activity converting the oxidized form of uracil to reduced form. Unfortunately, no such product was detected in the assays despite the attempts with various conditions. In addition to the possible deficiency of coenzymes, it was also likely that some intermediates could be the more suitable substrates of SSGG-03002 rather than the end product or uracil, so it is essential to expand the substrate selection if they are obtainable. Alternatively, we suspected that other proteins as partners of SSGG-03002 might be required to catalyze the reduction. Nonetheless, SSGG-03002 was clarified to be a player essential to the diversification of uridine-contained antibiotics, which was also manifested by its homologous protein NpsU. By coincidence, *SSGG-03002* gene was not found in the characterized BGCs of other uridyl peptide compounds, pacidamycins and sansanmycins, and accordingly, no reduced but only unsaturated double-bond at uracil C5-C6 was found in the structure of these compounds. SSGG-03002 was expected to be a useful tool in these strains to increase the product variety.

## Conclusions

In summary, mureidomycins derived from different amino acids and uracil are a subclass of uridyl peptide antibiotics with great potential against Gram-negative pathogens. Here we identified two unusual genes responsible for the modification of uracil ring. *SSGG-03002* encoding oxidoreductase and *SSGG-02980* encoding phosphatase are wide-spread in bacteria based on bioinformatics analyses. Gene disruption and compound structural determination verified that SSGG-03002 participates in the uracil C5–C6 double bond reduction, while SSGG-02980 has adverse effects. It was encouraging that disruption of *SSGG-03002* and *SSGG-02980* individually gave rise to the accumulation of specific MRDs and rMRDs, respectively, considerably facilitating the separation of the analogs with trivial structure differences. More profoundly, that SSGG-02980 predicted to be a phosphatase negatively regulates SSGG-03002 was unprecedented. Overall, these findings shed new light on the function and correlation of the two families of enzymes represented by SSGG-02980 and SSGG-03002, and provided a useful tool for diversification of uridyl peptide compounds to accelerate the discovery of this class of antibiotics.

## Methods

### Bacterial strains, plasmids and growth conditions

The bacterial strains and plasmids used in this study are listed in Additional file 3: Tables S1 and S2, and all primers are summarized in Additional file 3: Table S3. Previously, *S. roseosporus* NRRL 15998 (Sr-WT) was introduced with the exogenous activator gene *ssaA* for overexpression to activate the cryptic mureidomycin BGC [17], resulting in Sr-hA, which was used as the starting strain in the present work. *Escherichia coli* JM109 was used as a general host for propagating plasmids. *E. coli* ET12567 (pUZ8002) was used for conjugal transfer of plasmids into *Streptomyces* [22].

To harvest spores, Sr-WT and its derivatives were cultured on MM (0.05% L-Asn, 0.05% K_2_HPO_4_, 0.02% MgSO_4_·7H_2_O, 0.001% FeSO_4_·7H_2_O, 5% mannitol, 1% agar) for four days. To produce mureidomycins, Sr-WT or its derivatives were incubated in TSB (1.7% pancreatic digest of casein, 1.5% papaic digest of soybean, dextrose 0.25%, 0.5% NaCl) at 220 rpm for 2 days as seed culture, which was then transferred into ISP-2 at 1% of the medium volume for fermentation [17]. *E. coli* strains were grown in LB (1% tryptone, 0.5% yeast extract, 1% NaCl, 1% agar) at 37 °C for 15–20 h. When necessary, antibiotics were added to different media with final concentrations as follows: 100 μg/ml apramycin, 100 μg/ml kanamycin, 25 μg/ml chloramphenicol and 100 μg/ml hygromycin in LB medium for *E. coli*; 10 μg/ml apramycin, 50 μg/ml hygromycin and 25 μg/ml nalidixic acid in AS-1 medium for *S. roseosporus*.

### Construction of *ssaA* expression plasmid

To activate the mureidomycin BGC, *ssaA* expression plasmid was constructed. First of all, the ORF of *ssaA* was amplified with primer pair ssaAF/R from plasmid pSET152::P_*hrdB*_-*ssaA* [17], and the constitutive promoter P_*hrdB*_ from *Streptomyces coelicolor* was amplified with primer pair hrdF1/R1. Then, the two fragments were inserted into *Nde*I/*Xho*I-digested pIJ10500 with Gibson assembly to generate pJI10500::P_*hrdB*_-*ssaA* [23].

### Gene disruption and complementation

For disruption of gene *SSGG-02980,* the upstream and downstream DNA fragments of *SSGG-02980* were amplified by PCR with primer pairs 980upF/R and 980dnF/R using genomic DNA of Sr-WT as template. The two fragments were then ligated into *Eco*RV-digested pKC1139 with Gibson assembly to generate the disruption plasmid pKC1139-02980D, which was verified by PCR using the corresponding primer pairs 980crsF/R and 302crsF/R. To construct the *SSGG-02980* disruption mutant (∆02980-hA)*,* plasmid pKC1139-02980D was first introduced into *E. coli* ET12567 (pUZ8002) and subsequently transferred into Sr-WT via *E. coli*-*Streptomyces* conjugal transfer [22], followed by standard double-crossover mutant screening procedures [21]. The obtained mutant (∆02980) was verified by PCR with the primer pair 980crsF/R using genomic DNA as a template. Similarly, *SSGG-03002* gene was disrupted by using the disruption plasmid pKC1139-03002D to obtain strain (∆03002). For disruption of *SSGG-03002* in ∆02980, pKC1139-03002D was transferred into ∆02980 followed by the same screening procedures as for ∆03002 to generate mutant ∆02980/03002. Finally, pJI10500::P_*hrdB*_-*ssaA* was introduced into ∆02980, ∆03002 and ∆02980/03002 via conjugal transfer to obtain ∆02980-hA, ∆03002-hA and ∆02980/03002-hA, respectively.

To construct the *SSGG-02980* complementary plasmid pSET152::P_*hrdB*_-*02980*, the ORF of *SSGG-02980* was amplified by PCR using primer pair 980F/R, and P_*hrdB*_ promoter was amplified using primer pair hrdF2/R2 from *S. coelicolor*. The two fragments were then inserted into *Eco*RV-digested pSET152 with Gibson assembly to generate pSET152::P_*hrdB*_-*02980*. Finally, plasmid pSET152::P_*hrdB*_-*02980* was introduced into strain ∆02980-hA via conjugal transfer to generate the *SSGG-02980* complementary strain (∆02980c-hA).

The *SSGG-03002* complementary strain was constructed as follows: a DNA fragment containing *SSGG-03002* ORF, the 2.5 kb up- and downstream regions of *SSGG-03002* was amplified with primer pair 302uF1/302dR1 using genomic DNA of Sr-WT as template. This fragment was inserted into *Eco*RV-digested pKC1139 with T4 DNA ligase. Then the resultant plasmid pKC1139-03002c was transferred into ∆03002, and a general screening procedure similar to that for double-crossover mutant was employed to obtain the strain ∆03002c, which was verified by PCR using primer pair 302crsF/R. Subsequently, pJI10500::P_*hrdB*_-*ssaA* was introduced into ∆03002c to obtain the complementary strain ∆03002c-hA.

### HPLC and mass spectrometry analyses of mureidomycins

The HPLC analyses of mureidomycins in culture filtrates of Sr-hA and its derivative strains were carried out as previously described [17]. Ultrahigh pressure liquid chromatography-high resolution mass spectrometry (UPLC‒HRMS) and UPLC‒MS/MS analyses were carried out on Waters ACQUITY UPLC® and Xevo G2 Qtof MS systems (Waters Corporation, Milford, USA) equipped with an electrospray ion source and hybrid quadrupole-time-of-flight (Q-TOF) mass spectrometer in the MS^E^ model. The system was controlled with Masslynx V4.1. The ACQUITY UPLC BEH C_18_ Column (2.1 mm × 50 mm, 1.7 μm) was held at 45 °C. The mobile phase consisted of A and B with a flow rate of 0.3 ml/min, where A was water containing 0.1% formic acid and B was acetonitrile. The gradient elution program was set as follows: 0 min—100% A and 0% B; 10 min—85% A and 15% B; 15 min—50% A and 50% B; 17 min—0% A and 100% B; and 19 min—0% A and 100% B.

### Large-scale fermentation, isolation and structural determination of mureidomycin analogs

For isolation of mureidomycin analogs, three liters of fermentation broth of ∆02980-hA and ∆03002-hA were treated to obtain crude extracts as previously described [21]. After washing three times with *n*-hexane to remove the lipid, the crude extracts were subjected to preparative HPLC (Zorbax, SB-C18, 9.4 × 250 mm, 5 μm) with a flow rate of 2 ml/min at 280 nm detection wavelength. The gradient elution profile was set as acetonitrile in water from 10 to 32% in 20.0 min (0–20 min) followed by 100% acetonitrile for 5 min (20.1–25.1 min) and then 100% water for 5 min (25.1–30.1 min). The NMR spectra were recorded on a 500 MHz Bruker spectrometer using methanol-d4 as the solvent.

### RNA isolation and real-time quantitative PCR (RT-qPCR)

*S. roseosporus* and its derivative strains were fermented, and samples were taken at different time points for RNA isolation. RT-qPCR was performed according to the method described previously [21].

### Supplementary Information

**Additional file 1.** The table of the homologous proteins of SSGG-03002 obtained by using BLASTP program.

**Additional file 2.** The table of the homologous proteins of SSGG-02980 obtained by using BLASTP program.

**Additional file 3.** **Table S1**. Strains used in this study. **Table S2**. Plasmids used in this study. **Table S3**. Primers used in this study. **Figure S1**. Verification of *SSGG-02980* and/or *03002* disruption mutants and the complementary strains. Agarose gel electrophoresis of the PCR products for verification of *SSGG-02980* and/or *03002* gene disruption mutants and their complementary strains. M, DL2000 DNA marker. Lanes 1, 3, 5, 7, 9 and 11, PCR analyses of *SSGG-02980* in Sr-hA, Δ02980-hA, Δ02980c-hA, Δ03002-hA, Δ03002c-hA and Δ02980/03002-hA. Lanes 2, 4, 6, 8, 10 and 12, PCR analyses of *SSGG-03002* in Sr-hA, Δ02980-hA, Δ02980c-hA, Δ03002-hA, Δ03002c-hA and Δ02980/03002-hA. In the gene disruption mutants, no PCR products were obtained for the relevant genes, while the expected bands were shown in the complementory strains, verifying the correctness of the strains. **Figure S2**. UPLC-HRMS analyses of mureidomycins in Δ03002-hA. **A**, EIC spectra of MRDs (**1-6**) (top) and rMRDs (**1’-6’**) (bottom) in Δ03002-hA. Only MRDs were produced but **1’-6’** were not detected. **B**, Mass spectra of the extracted quasi-molecular ions of mureidomycins analogues **1-6**. **Figure S3**. UPLC-HRMS analyses of mureidomycins in Δ02980-hA. **A**, EIC spectra of MRDs (**1-6**) (top) and rMRDs (**1’-6’**) (bottom) in Δ02980-hA. **B**, Mass spectra of the extracted quasi-molecular ions of MRDs and rMRDs (**1, 3-6** and **1’, 3’-6’**). The extracted MS for **2** (*m/z* 899.3247) and **2’** (*m/z* 901.3372) were not observed due to too low abundance. Compound **1’, 3’ -6’** were accumulated, while the production of **1-6** was significantly reduced. **Figure S4.** UPLC-HRMS analyses of mureidomycins in Δ02980c-hA. **A**, EIC spectra of MRDs (**1-6**) (top) and rMRDs (**1’-6’**) (bottom) in Δ02980c-hA. **B**, Mass spectra of the extracted quasi-molecular ions of **1-6** and **1’-6’**. The production of both MRDs and rMRDs was restored. **Figure S5.** UPLC-HRMS analyses of mureidomycins in Δ03002c-hA. **A**, EIC spectra of MRDs (**1-6**) (top) and rMRDs (**1’-6’**) (bottom) in Δ03002-hA. **B**, Mass spectra of the extracted quasi-molecular ions of mureidomycins analogues **1, 3-6** and **1’, 3’-6’**. The extracted MS for **2** (*m/z* 899.3245) and **2’** (*m/z* 901.3394) were not observed due to too low abundance. **Figure S6**. UPLC-HRMS analyses of mureidomycins in Δ02980/03002-hA. **A**, EIC spectra of MRDs (**1-6**) (top) and rMRDs (**1’-6’**) (bottom) in Δ02980/03002-hA. Only MRDs (**1-6**) were produced but rMRDs (**1’-6’**) were not detected. **B**, Mass spectra of the extracted quasi-molecular ions of **1-6**. **Table S4**. NMR data for component (**3**) in CD3OD. a, ^13^C NMR data were assigned based on combination of the HMBC correlations and the comparison with that of *N*-acetylmureidomycin E and G. Abbreviations for the structure units: *m*-Tyr (*m*-tyrosine), AMBA (2-amino-3-methylaminobutyric acid). **Table S5**. NMR data for component (**4**) in CD3OD. a, ^13^C NMR data were assigned based on combination of the HMBC correlations and the comparison with that of *N*-acetylmureidomycin E and G. Abbreviations for the structure units: *m*-Tyr (*m*-tyrosine), AMBA (2-amino-3-methylaminobutyric acid). **Table S6**. NMR data for component (**5**) in CD3OD. a, ^13^C NMR data were assigned based on combination of the HMBC correlations and the comparison with that of N-acetylmureidomycin E. Abbreviations for the structure units: m-Tyr (m-tyrosine), AMBA (2-amino-3-methylaminobutyric acid). **Table S7**. NMR data for component (**3’**) in CD3OD. a, ^13^C NMR data were assigned based on combination of the HMBC correlations and the comparison with that of *N*-acetylmureidomycin E and G. Abbreviations for the structure units: *m*-Tyr (*m*-tyrosine), AMBA (2-amino-3-methylaminobutyric acid). **Table S8**. NMR data for component (**4’**) in CD3OD. a, ^13^C NMR data were assigned based on the combination of HMBC correlations and the comparison with that of *N*-acetylmureidomycin E and G. Abbreviations for the structure units: *m*-Tyr (*m*-tyrosine), AMBA (2-amino-3-methylaminobutyric acid). **Figure S7**. ^1^H NMR spectrum (500 MHz) of component (**3**) in CD3OD. **Figure S8**. ^13^C NMR spectrum (125 MHz) of component (**3**) in CD3OD. **Figure S9**. Dept135 spectrum of component (**3**) in CD3OD. **Figure S10**. COSY spectrum of component (**3**) in CD3OD. **Figure S11**. HSQC spectrum of component (**3**) in CD3OD. **Figure S12**. HMBC spectrum of component (**3**) in CD3OD. **Figure S13**. ^1^H NMR spectrum (500 MHz) of component (**4**) in CD3OD. **Figure S14**. ^13^C NMR spectrum (125 MHz) of component (**4**) in CD3OD. **Figure S15**. Dept135 spectrum of component (**4**) in CD3OD. **Figure S16**. COSY spectrum of component (**4**) in CD3OD. **Figure S17**. HSQC spectrum of component (**4**) in CD3OD. **Figure S18**. HMBC spectrum of component (**4**) in CD3OD. **Figure S19**. ^1^H NMR spectrum (500 MHz) of component (**5**) in CD3OD. **Figure S20**. ^13^C NMR spectrum (125 MHz) of component (**5**) in CD3OD. **Figure S21**. Dept135 spectrum of component (**5**) in CD3OD. **Figure S22**. COSY spectrum of component (**5**) in CD3OD. **Figure S23.** HSQC spectrum of component (**5**) in CD3OD. **Figure S24**. HMBC spectrum of component (**5**) in CD3OD. **Figure S25**. ^1^H NMR spectrum (500 MHz) of component (**3’**) in CD3OD. **Figure S26**. ^13^C NMR spectrum (125 MHz) of component (**3’**) in CD3OD. **Figure S27**. Dept135 spectrum of component (**3’**) in CD3OD. **Figure S28.** COSY spectrum of component (**3’**) in CD3OD. **Figure S29.** HSQC spectrum of component (**3’**) in CD3OD. **Figure S30.** HMBC spectrum of component (**3’**) in CD3OD. **Figure S31.** ^1^H NMR spectrum (500 MHz) of component (**4’**) in CD3OD. **Figure S32.** ^13^C NMR spectrum (125 MHz) of component (**4’**) in CD3OD. **Figure S33.** Dept135 spectrum of component (**4’**) in CD3OD. **Figure S34.** COSY spectrum of component (**4’**) in CD3OD. **Figure S35.** HSQC spectrum of component (**4’**) in CD3OD. **Figure S36.** HMBC spectrum of component (**4’**) in CD3OD. **Figure S37**. ^1^H NMR spectrum (500 MHz) of component (**5’**) in CD3OD. **Figure S38.** ^13^C NMR spectrum (125 MHz) of component (**5’**) in CD3OD. **Figure S39.** Putative structure of compound **2’** and its isomer.

**Additional file 4.** Expression of SSGG-03002 and SSGG-02980 in *E. coli*. Protein sequencing and modification analysis by mass spectrometry. **Figure S40**. Effect of *SSGG-02980* disruption on the transcription of *SSGG-03002*. Black, Sr-hA; Red, Δ02980-hA; Blue, Δ02980c-hA. The transcription of *SSGG-03002* was elevated in *SSGG-02980* disruption mutant (Δ02980-hA), suggesting that SSGG-02980 could have repressive roles on the transcription of *SSGG-03002*; while, rather unexpectedly, the transcription in the complementary strain Δ02980c-hA did not return to the level as in starting strain Sr-hA, implying that SSGG-02980 might affect SSGG-03002 via multiple ways either directly or indirectly. So the inhibition on *SSGG-03002* transcription could be just one of them. **Figure S41.** Expression of *SSGG-03002* with or without *SSGG-02980*. **A**, Verification of the recombinant strains for overexpression of *SSGG-03002* with or without *SSGG-02980* in *E. coli* C41 (DE3). Lane 1, plasmid pET28a::*02980* as reference; Lane 2, plasmids extracted from C41/03002+02980; Lane 3, the plasmid extracted from C41/03002. Lane 4, plasmid pET23b::*03002* as reference. M, DNA ladder. **B**, SDS-PAGE analysis of the purified recombinant proteins, SSGG-03002 and SSGG-02980. Lane 1 and 2, SSGG03002 purified from strains C41/03002 and C41/03002+02980, respectively. In the latter, SSGG-02980 was co-purified with SSGG-03002 by using his-tag affinity column. M, protein standard. **Figure S42**. Phosphorylation of SSGG-03002 in the absence (black bar) or presence (grey bar) of SSGG-02980. The protein samples were digested following standard procedures (see Method S1 in the Additional file 4), and then subjected to LC-MS analysis for protein sequencing. The phosphorylated peptide fragments with good reproducibility in each sample were chosen for the calculation. The phosphorylation level at T304, S302 and Y305 of recombinant SSGG-03002 co-expressed with SSGG-02980 was lower than that expressed alone. However, statistic analysis indicated that the difference was not significant (*P* > 0.05), so SSGG-02980 does not have considerable effect on the phosphorylation of SSGG-03002 derived from *E. coli*. Means and standard errors were calculated with the data from three independent experiments. **Figure S43.** Alignment of SSGG-03002 with NpsU. The alignment was done with BLASTP programme, and the identity was determined to be 94.19%. The accession number of NpsU from *Streptomyces sp.* DSM 5940 in GenBank is ADY76683.1.

## Data Availability

All data generated or analyzed during this study are included in this published article and supplementary information files.
