# Supplementary material for: New insights into the dihydro-mureidomycin biosynthesis controlled by two unusual proteins in Streptomyces roseosporus
Source: Microb Cell Fact. 2023 Dec 12;22:255. doi: 10.1186/s12934-023-02260-6 (PMC10714638; doi:10.1186/s12934-023-02260-6)
Supplement: Supplementary file 3 — Additional file 3. Table S1. Strains used in this study. Table S2. Plasmids used in this study. Table S3. Primers used in this study. Figure S1. Verification of SSGG-02980 and/or 03002 disruption mutants and the complementary strains. Agarose gel electrophoresis of the PCR products for verification of SSGG-02980 and/or 03002 gene disruption mutants and their complementary strains. M, DL2000 DNA marker. Lanes 1, 3, 5, 7, 9 and 11, PCR analyses of SSGG-02980 in Sr-hA, Δ02980-hA, Δ02980c-hA, Δ03002-hA, Δ03002c-hA and Δ02980/03002-hA. Lanes 2, 4, 6, 8, 10 and 12, PCR analyses of SSGG-03002 in Sr-hA, Δ02980-hA, Δ02980c-hA, Δ03002-hA, Δ03002c-hA and Δ02980/03002-hA. In the gene disruption mutants, no PCR products were obtained for the relevant genes, while the expected bands were shown in the complementory strains, verifying the correctness of the strains. Figure S2. UPLC-HRMS analyses of mureidomycins in Δ03002-hA. A, EIC spectra of MRDs (1-6) (top) and rMRDs (1’-6’) (bottom) in Δ03002-hA. Only MRDs were produced but 1’-6’ were not detected. B, Mass spectra of the extracted quasi-molecular ions of mureidomycins analogues 1-6. Figure S3. UPLC-HRMS analyses of mureidomycins in Δ02980-hA. A, EIC spectra of MRDs (1-6) (top) and rMRDs (1’-6’) (bottom) in Δ02980-hA. B, Mass spectra of the extracted quasi-molecular ions of MRDs and rMRDs (1, 3-6 and 1’, 3’-6’). The extracted MS for 2 (m/z 899.3247) and 2’ (m/z 901.3372) were not observed due to too low abundance. Compound 1’, 3’ -6’ were accumulated, while the production of 1-6 was significantly reduced. Figure S4. UPLC-HRMS analyses of mureidomycins in Δ02980c-hA. A, EIC spectra of MRDs (1-6) (top) and rMRDs (1’-6’) (bottom) in Δ02980c-hA. B, Mass spectra of the extracted quasi-molecular ions of 1-6 and 1’-6’. The production of both MRDs and rMRDs was restored. Figure S5. UPLC-HRMS analyses of mureidomycins in Δ03002c-hA. A, EIC spectra of MRDs (1-6) (top) and rMRDs (1’-6’) (bottom) in Δ03002-hA. B, Mass spectra of the extract [file 12934_2023_2260_MOESM3_ESM.pdf]

# Additional file 3

**New insights into the dihydro-mureidomycin biosynthesis controlled by two unusual proteins in *Streptomyces roseosporus***

**Ning Liu[1†], Yang Xu[1,2†], Fei Shang[3], Huiying Sun[1,2], Xiang Liu[1,2], Ying Huang[1], HuarongTan[1,2*], Jihui Zhang[1*]**

**Affiliations:**

[1] State Key Laboratory of Microbial Resources, Institute of Microbiology, Chinese Academy of Sciences, Beijing 100101, China.

[2] College of Life Sciences, University of Chinese Academy of Sciences, Beijing 100049, China.

[3] Analytical and Testing Center, Beijing University of Chemical Technology, Beijing 100029, China.

Table S1 Strains used in this study

| Strains | Description/Genotype | Source/Ref. |
|---|---|---|
| *Streptomyces roseosporus* NRRL 15998 | Wild type strain (Sr-WT) | Board Institute |
| Sr-hA | Sr-WT/pIJ10500::P$_{hrdB}$-*ssaA* | This work |
| Δ02980-hA | *SSGG-02980* disrupted mutant of Sr-hA | This work |
| Δ03002-hA | *SSGG-03002* disrupted mutant of Sr-hA | This work |
| Δ02980c-hA | Δ02980-hA/pSET152::P$_{hrdB}$-*02980* | This work |
| Δ03002c-hA | *SSGG-03002 in situ* complemented mutant of Δ03002-hA | This work |
| Δ02980/03002-hA | Both *SSGG-02980* and *03002* disrupted mutant of Sr-hA | This work |
| *Escherichia coli* (*E. coli*) | | |
| JM109 | F', *proA*$^+$*B*$^+$, *lacI*$^q$, Δ*(lacZ)M15/*Δ*(lac-proAB)*, *gyrA96*, *recA1*, *relA1*, *endA1*, *hsdR17* | Invitrogen |
| C41 (DE3) | F$^-$, *ompT*, *gal dcm hsd S$_B$* (r$_B$$^-$, m$_B$$^-$) (DE3) | Lucigen |
| ET12567/pUZ8002 | *dam*$^-$ *dcm*$^-$ *hsdM*$^-$ pUZ8002 | (Kieser et al., 2000) |
| C41/03002 | C41(DE3) derivative for expression of *SSGG-03002* containing pET23b::*03002* | This work |
| C41/03002+02980 | C41(DE3) derivative for expression of *SSGG-03002* and *-02980* containing both pET23b::*03002* and pET28a::*02980* | This work |

Table S2 Plasmids used in this study

| Plasmids | Description | Source/Ref. |
|---|---|---|
| pSET152 | *aac(3)IV*, *lacZ*, *rep*$^{pMB1*}$*att*$\phi$*C31*, *oriT*; | (Kieser et al., 2000) |
| pKC1139 | *aac(3)IV*, *E. coli-Streptomyces* shuttle plasmid contains a *Streptomyces* temperature-sensitive origin of replication | (Kieser et al., 2000) |
| pIJ105000 | $Hyg^r$, a derivative of pMS82 containing φBT1 integrase coding gene | (Gregory et al., 2003) |
| pKC1139-02980D | pKC1139 derivative, used for disruption of *SSGG-02980* | This work |
| pKC1139-03002D | pKC1139 derivative, used for disruption of *SSGG-03002* | This work |
| pIJ10500::$P_{hrdB}$-*ssaA* | pIJ10500 containing $P_{hrdB}$ of *S. coelicolor* A3(2) and *ssaA*, used for expression of *ssaA* | This work |
| pSET152::$P_{hrdB}$-*02980* | pSET152 containing $P_{hrdB}$ and *SSGG-02980*, used for complementation of *SSGG-02980* | This work |
| pKC1139-03002c | pKC1139 derivative, used for complementation of *SSGG-03002* | This work |
| pET23b::*03002* | pET23b derivative, used for expression of *SSGG-03002* in *E. coli* | This work |
| pET28a::*02980* | pET28a derivative, used for expression of *SSGG-02980 in E. coli* | This work |

Table S3 Primers used in this study

| primers | sequence (5′-3′) | description |
|---|---|---|
| 980upF | gatccgcggccgcgcgcgatgcagctccacctgtccacc | pKC1139-02980D construction |
| 980upR | ccagaactgaggcgtagtaatcg | pKC1139-02980D construction |
| 980dnF | cgattactacgcctcagttctggccgagtccgaccattcttattg | pKC1139-02980D construction |
| 980dnR | gacatgattacgaattcgatcgaggtcaatgagcagggtg | pKC1139-02980D construction |
| 302upF | gatccgcggccgcgcgcgatccctgggtatcgccgctc | pKC1139-03002D construction |
| 302upR | cctgttctccaccgtgctca | pKC1139-03002D construction |
| 302dnF | agcacggtggagaacagggttggacctgagctggcatgt | pKC1139-03002D construction |
| 302dnR | gacatgattacgaattcgatagcacgatacccctccggtg | pKC1139-03002D construction |
| 980crsF | gacgtactgtgcctgcaagag | Verification of *SSGG-02980* gene disruption strain |
| 980crsR | actcacctggcaataagaatgg | Verification of *SSGG-02980* gene disrupton strain |
| 302crsF | gagcacggtggagaacagga | Verification of *SSGG-03002* gene disrupton strain |
| 302crsR | cagcgacgccagcacatag | Verification of *SSGG-03002* gene disrupton strain |
| ssaAF | tcaacgttccgagaggttgttcatgaataccatgaccgaatccg | pIJ10500::$P_{hrdB}$-*ssaA* construction |
| ssaAR | gcggccgcgcgcgatcatattcaggcacattgtgccctc | pIJ10500::$P_{hrdB}$-*ssaA* construction |
| hrdF1 | cgtggtccttgtagtcc | Constuction of $P_{hrdB}$ in pIJ10500 |
| hrdR1 | gaacaacctctcggaacgttga | Constuction of $P_{hrdB}$ in pIJ10500 |
| hrdF2 | gatccgcggccgcgcgcgat*ccgccttccgccggaacg* | Constuction of $P_{hrdB}$ in pSET152 |
| hrdR2 | gaacaacctctcggaacgttga | Constuction of $P_{hrdB}$ in pSET152 |
| 980F | tcaacgttccgagaggttgttcatgaggcctacgctgctga | Construction of pSET152::$P_{hrdB}$-*02980* for *SSGG-02980* complementation |
| 980R | gacatgattacgaattcgatttctcgggtgcccacaac | Construction of pSET152::$P_{hrdB}$-*02980* for *SSGG-02980* complementation |
| 302uF1 | tcgccgctcatacaccca | Construction of pKC1139-03002c for *SSGG-03002* complementation |
| 302dR1 | agtcccaacaaccgcccac | Construction of pKC1139-03002c for *SSGG-03002* complementation |
| 03002-F | ctttaagaaggagatatacagtgcagcgaactcatgagt | Construction of pET23b::*03002* |
| 03002-R | tcagtggtggtggtggtggtgcgtgccctcggctctgaccgaac | Construction of pET23b::*03002* |
| 02980-F | atgggtcgcggatccgaattcatgaggcctacgctg | Construction of pET28a::*02980* |
| 02980-R | tgcggccgcaagcttctacaccagcacact | Construction of pET28a::*02980* |
| 3002-rtF | gagcgtcacgccggt | For RT-qPCR |
| 3002-rtR | acttcgaagctcacttgactgcc | For RT-qPCR |
| 16S-rtF | tcatgccccttatgtcttggg | For RT-qPCR |
| 16S-rtR | ccggctttttgagattcgctc | For RT-qPCR |

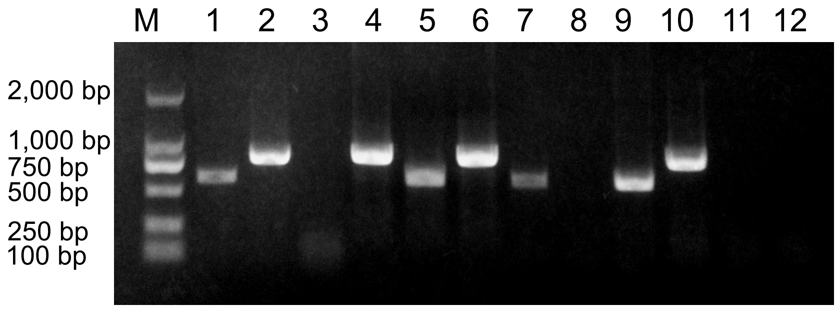


**Figure S1 Verification of *SSGG-02980* and/or *03002* disruption mutants and the complementary strains**

Agarose gel electrophoresis of the PCR products for verification of *SSGG-02980* and/or *03002* gene disruption mutants and their complementary strains. M, DL2000 DNA marker. Lanes 1, 3, 5, 7, 9 and 11, PCR analyses of *SSGG-02980* in Sr-hA, Δ02980-hA, Δ02980c-hA, Δ03002-hA, Δ03002c-hA and Δ02980/03002-hA. Lanes 2, 4, 6, 8, 10 and 12, PCR analyses of *SSGG-03002* in Sr-hA, Δ02980-hA, Δ02980c-hA, Δ03002-hA, Δ03002c-hA and Δ02980/03002-hA. In the gene disruption mutants, no PCR products were obtained for the relevant genes, while the expected bands were shown in the complementory strains, verifying the correctness of the strains.

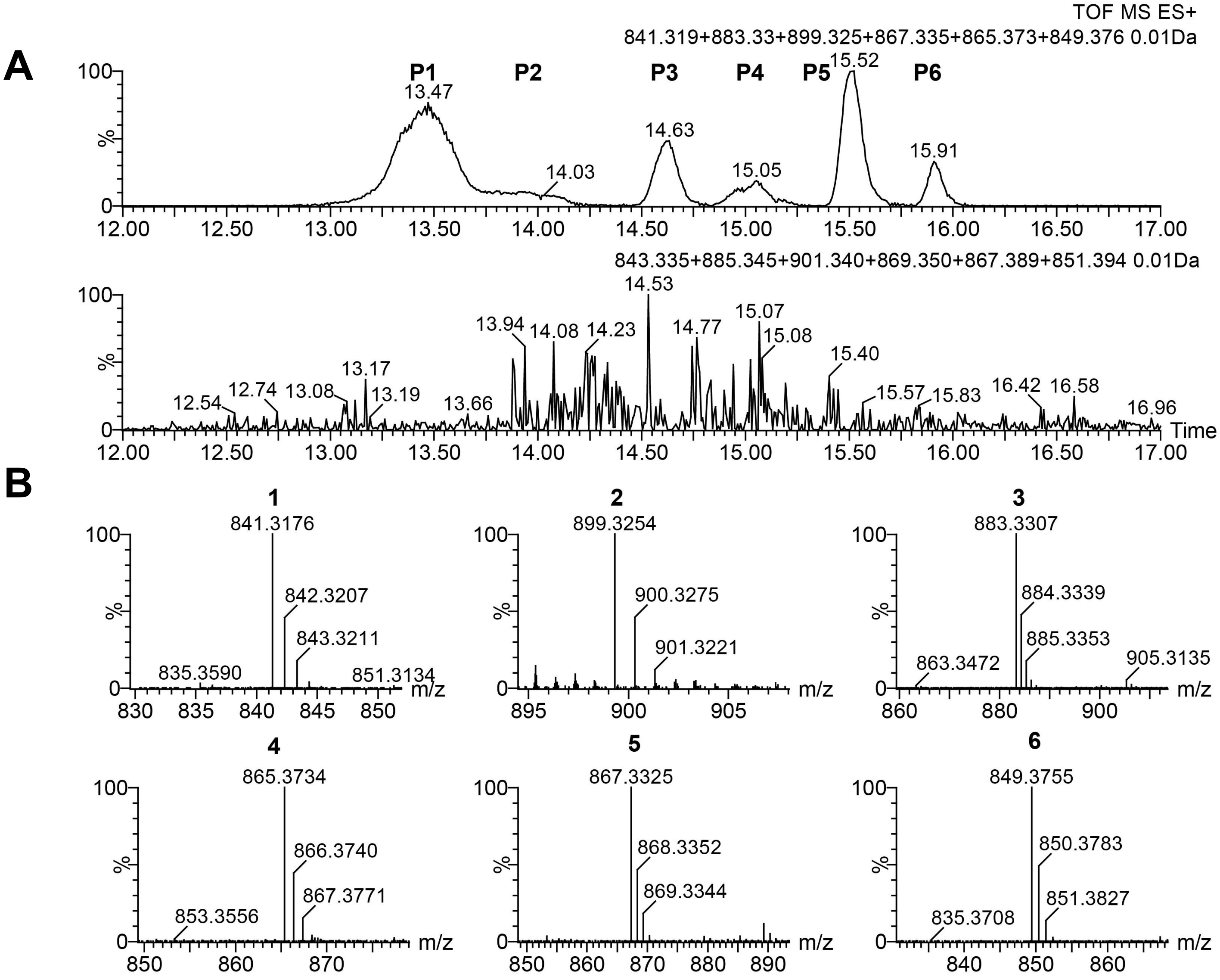


**Figure S2. UPLC-HRMS analyses of mureidomycins in Δ03002-hA.**

**A**, EIC spectra of MRDs (**1-6)** (top) and rMRDs (**1'-6')** (bottom) in Δ03002-hA. Only MRDs were produced but **1'-6'** were not detected. **B**, Mass spectra of the extracted quasi-molecular ions of mureidomycins analogues **1-6**.

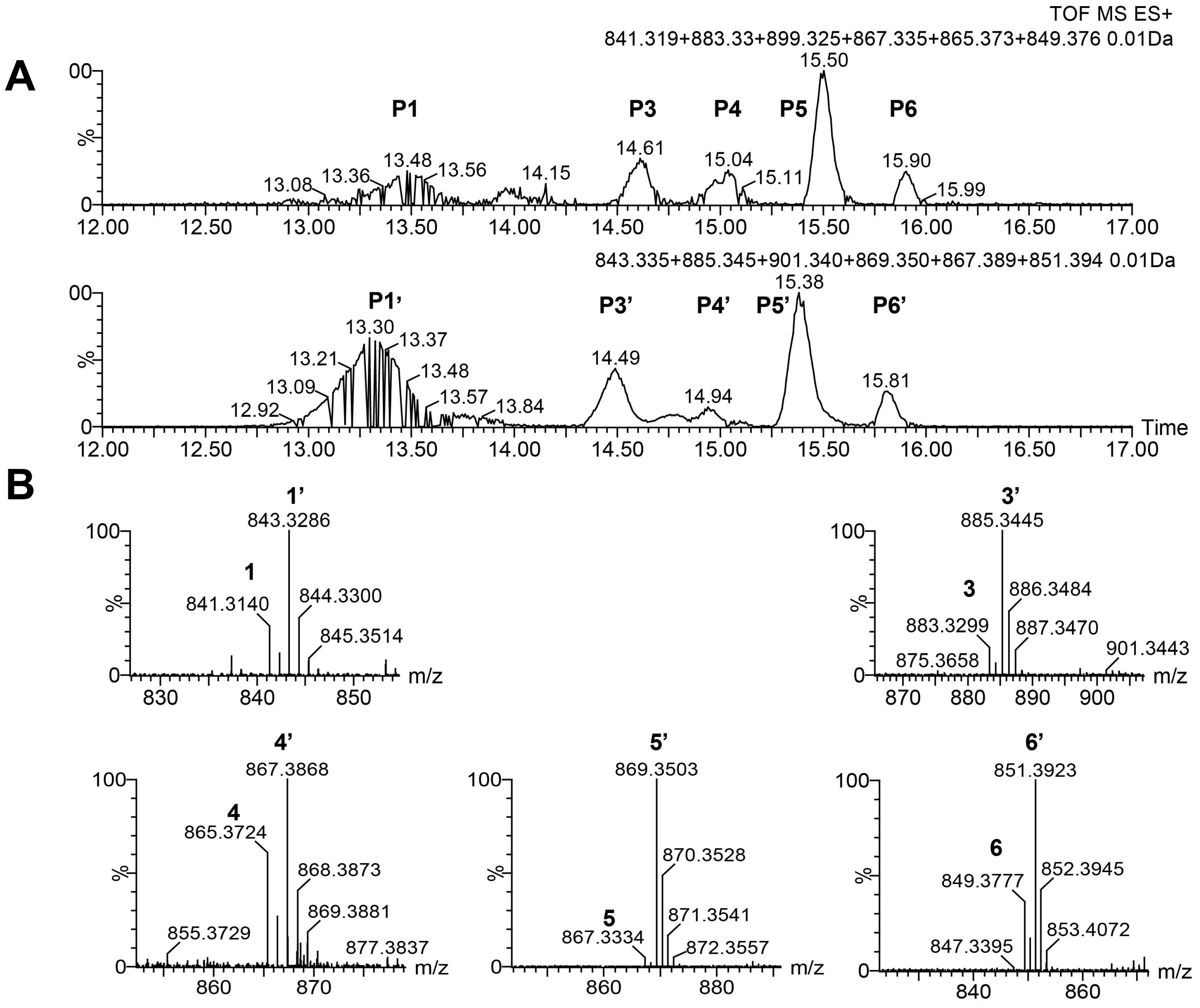


**Figure S3. UPLC-HRMS analyses of mureidomycins in Δ02980-hA.**

**A,** EIC spectra of MRDs (**1-6**) (top) and rMRDs **(1'-6')** (bottom) in Δ02980-hA. **B**, Mass spectra of the extracted quasi-molecular ions of MRDs and rMRDs (**1, 3-6** and **1', 3'-6'**). The extracted MS for **2** (*m/z* 899.3247) and **2'** (*m/z* 901.3372) were not observed due to too low abundance. Compound **1', 3' -6'** were accumulated, while the production of **1-6** was significantly reduced.

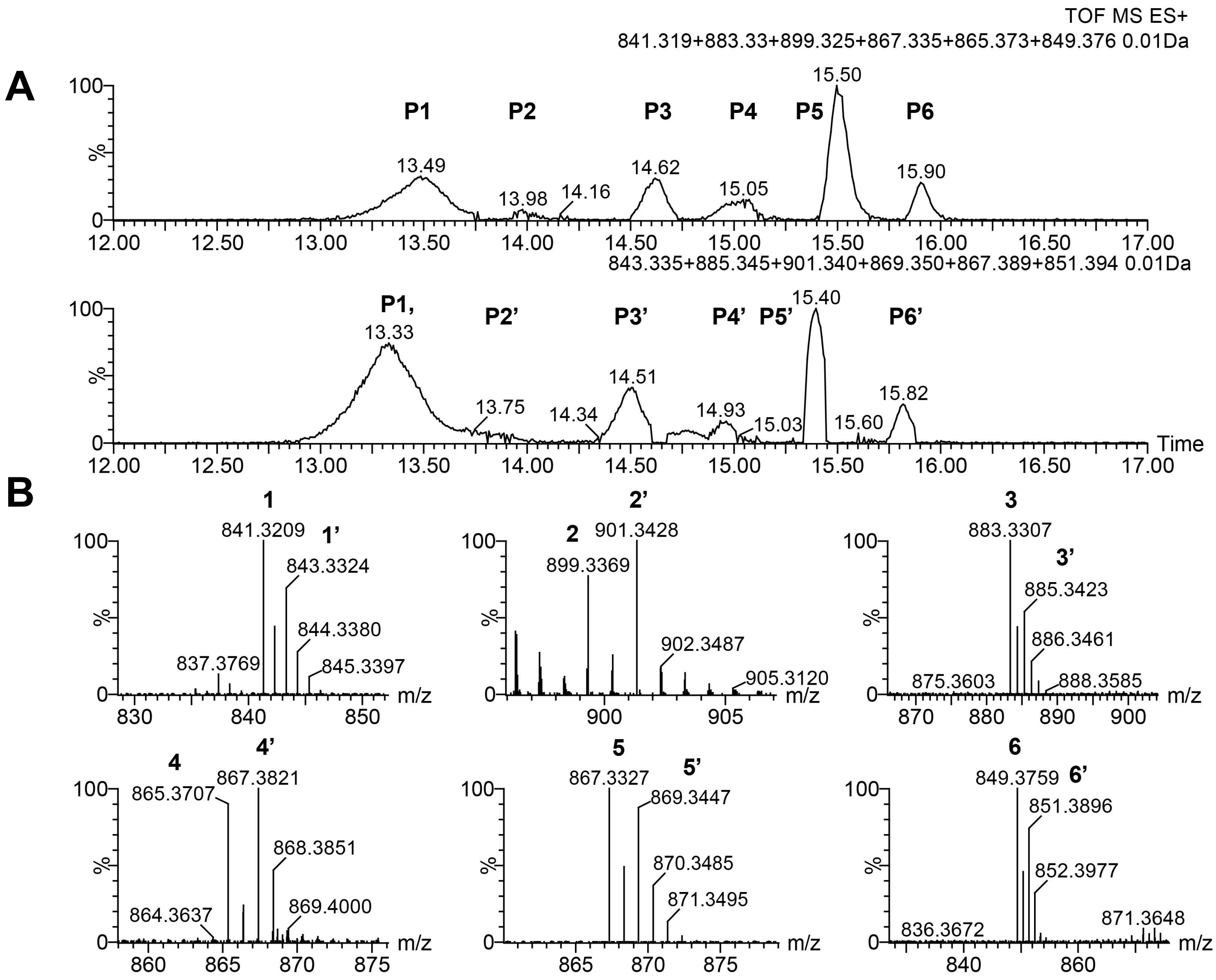


**Figure S4. UPLC-HRMS analyses of mureidomycins in Δ02980c-hA.**

**A,** EIC spectra of MRDs (**1-6)** (top) and rMRDs **(1'-6')** (bottom) in Δ02980c-hA. **B**, Mass spectra of the extracted quasi-molecular ions of **1-6** and **1'-6'**. The production of both MRDs and rMRDs was restored.

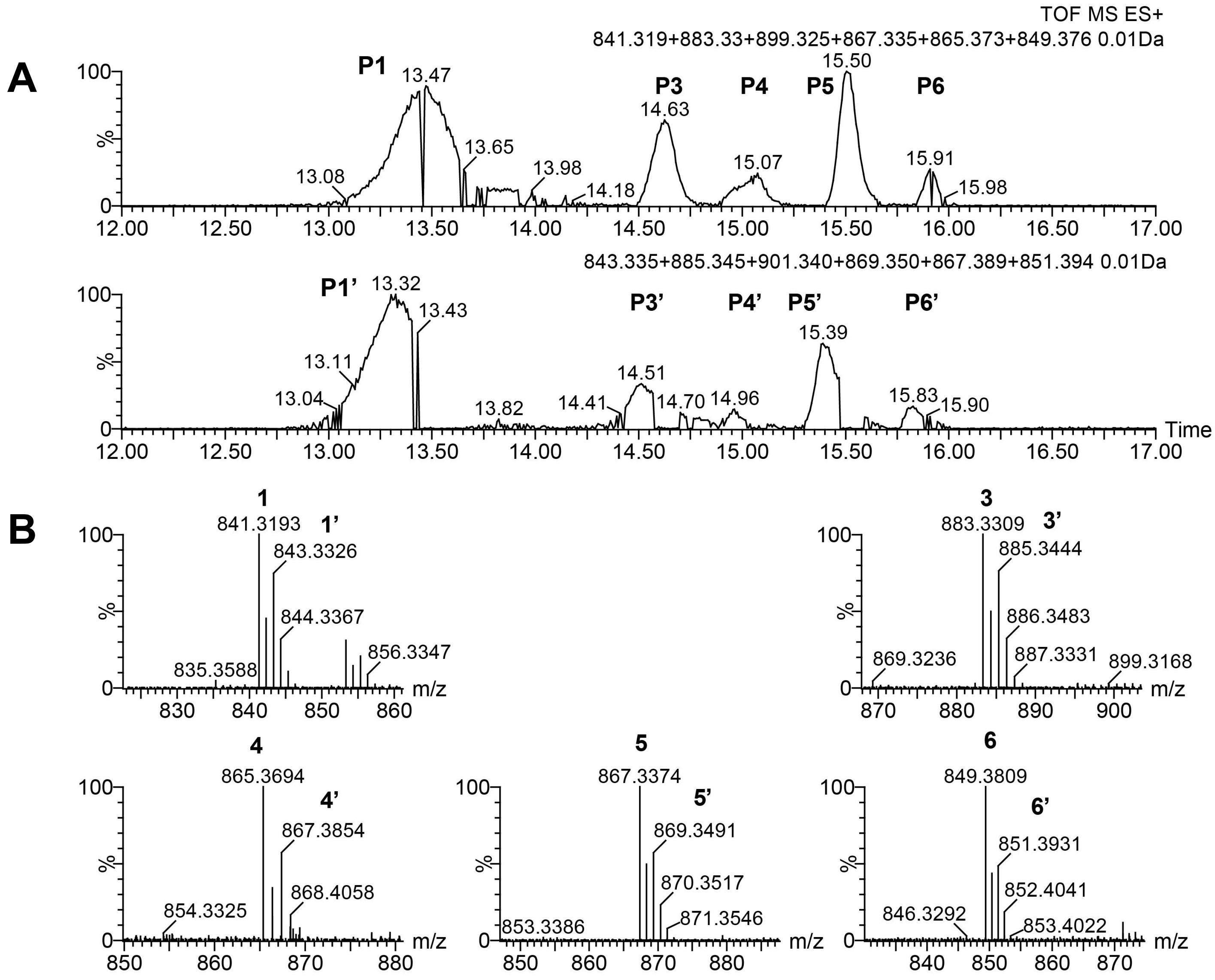


**Figure S5. UPLC-HRMS analyses of mureidomycins in Δ03002c-hA.**

**A,** EIC spectra of MRDs (**1-6)** (top) and rMRDs **(1'-6')** (bottom) in Δ03002-hA. **B**, Mass spectra of the extracted quasi-molecular ions of mureidomycins analogues **1, 3-6 and 1', 3'-6'**. The extracted MS for **2** (*m/z* 899.3245) and **2'** (*m/z* 901.3394) were not observed due to too low abundance.

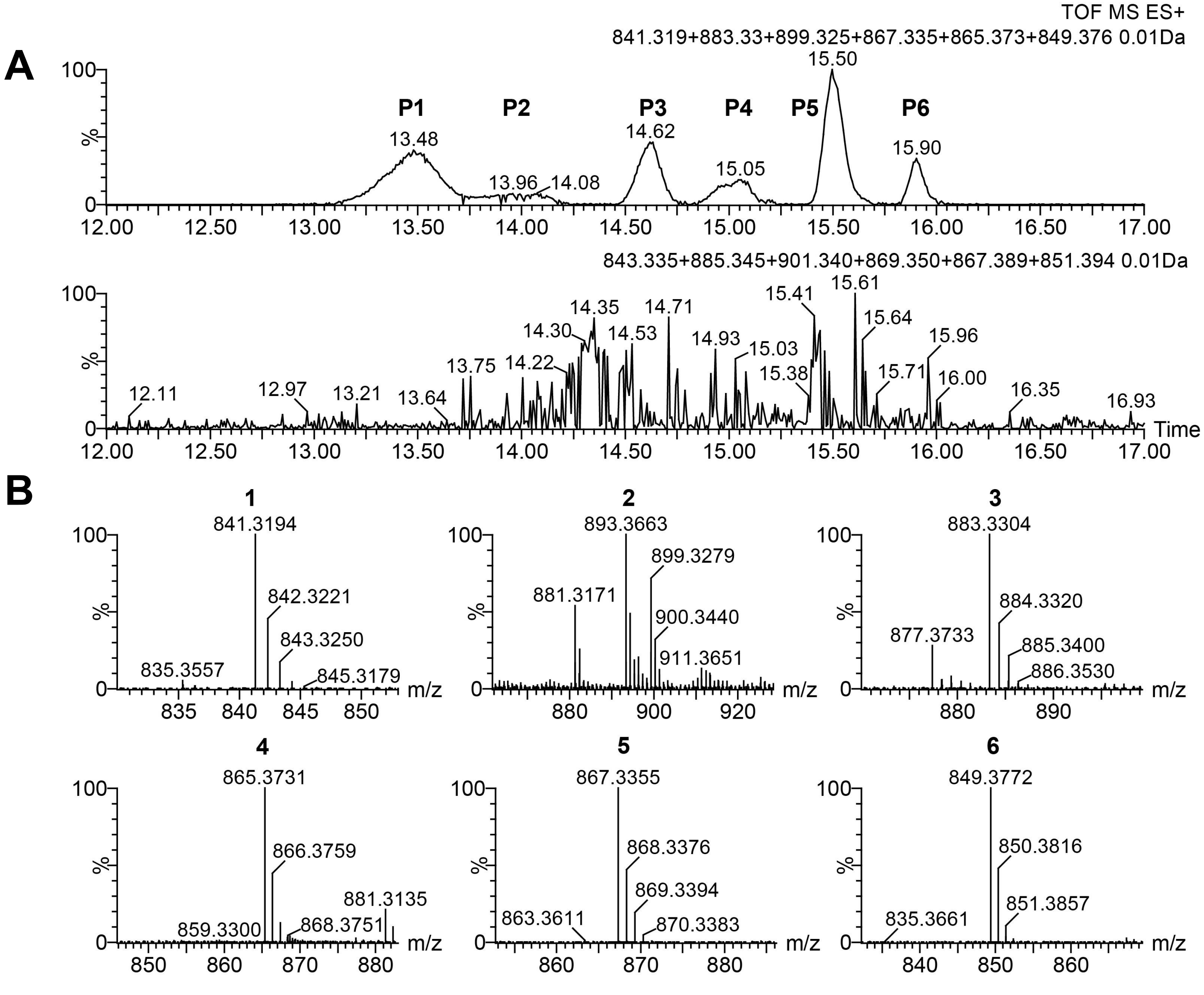


**Figure S6. UPLC-HRMS analyses of mureidomycins in Δ02980/03002-hA.**

**A,** EIC spectra of MRDs (**1-6**) (top) and rMRDs **(1'-6')** (bottom) in Δ02980/03002-hA. Only MRDs (**1-6**) were produced but rMRDs (**1'-6')** were not detected. **B**, Mass spectra of the extracted quasi-molecular ions of **1-6**.

Table S4 NMR data for component (**3**) in $CD_3OD$.

| Position | *N*-acetylmyreidomycin A | | | | iso-*N*-acetylmyreidomycin A (**3**$_{iso}$) | |
|---|---|---|---|---|---|---|
| | $\delta_C$-ppm | $\delta_H$-ppm (*J*, Hz) | COSY | Key HMBC | $\delta_C$-(ppm) | $\delta_H$-ppm (*J*, Hz) |
| Uracil-2 | 152.1, N-C=O | | | | 152.1 | |
| Uracil-4 | 165.9, N-C=O | | | | 166.1 | |
| Uracil-5 | 103.5, CH | 5.59 (d, 8.0) | Uracil-H6 | Uracil-C4, C6 | 103.4 | 5.76 (d, 8.0) |
| Uracil-6 | 141.2, CH | 7.12 (d, 8.0) | Uracil-H5 | Uracil-C2, C4, C5; Sugar-C1 | 142.0 | 7.41 (d, 8.0) |
| Sugar-1 | 94.5, O-CH-N | 6.05 (d, 3.0) | Sugar-H2 | Sugar-C2, C3 (w), C4; Uracil-C2, C6 | 95.2 | 6.03 (d, 2.0) |
| Sugar-2 | 73.9, O-CH | 4.36 (m) | Sugar-H1, H3 | Sugar-C4 | 74.1 | 4.49 (m) |
| Sugar-3 | 34.9, $CH_2$ | 2.61 (m)/2.92 (m) | Sugar-H2, H5 | Sugar-C1, C2, C4, C5 | 35.0 | 2.61 (m)/2.92 (m) |
| Sugar-4 | 143.7, >C= | | | | 144.2 | |
| Sugar-5 | 98.3, -CH= | 5.98 (s) | Sugar-H3 | Sugar-C3, C4; AMBA-C1 | 98.0 | 5.93 (s) |
| AMBA-1 | 168.3, C=O | | | | 168.1 | |
| AMBA-2 | 56.8, CH | 4.70 (m) | AMBA-H3 | AMBA-C1, C3, C4; Met-C1 | 57.3 | 4.68 (m) |
| AMBA-3 | 52.5, CH | 4.91 (m) | AMBA-H2, H4 | None/AMBA-C1, C2, C4, C6; m-Tyr-AC1 | 55.6 | 4.17 (m) |
| AMBA-4 | 14.2, $CH_3$ | 1.19 (d, 6.5) | AMBA-H3 | AMBA-C2, C3 | 14.5 | 0.74 (d, 6.5) |
| AMBA-6 | 31.1, N-$CH_3$ | 3.03 (s) | None | AMBA-C3; *m*-Tyr-AC1 | 28.8 | 2.66 (s) |
| Met-1 | 175.0, N-C=O | | | | 174.8 | |
| Met-2 | 54.3, CH | 4.36 (m) | Met-H3 | Met-C1, C3, C4; Ureido | 54.3 | 4.36 (m) |
| Met-3 | 33.5, $CH_2$ | 1.82 (m)/1.98 (m) | Met-H2, H4 | Met-C1, C2, C4 | 33.4 | 1.82 (m)/1.98 (m) |
| Met-4 | 31.1, S-$CH_2$ | 2.46 (m) | Met-H3 | Met-C2, C3, C6 | 31.1 | 2.46 (m) |
| Met-6 | 15.4, $CH_3$ | 2.03 (s) | None | Met-C4 | 15.4 | 2.03 (s) |
| Ureido | 159.7, N-C=O | | | | 159.7 | |
| *m*-Tyr-A1 | 174.2, N-C=O | | | | 173.6 | |
| *m*-Tyr-A2 | 52.5, CH | 4.94 (dd, 4.0, 10.0) | *m*-Tyr-AH3 | *m*-Tyr-AC1, C3, C4 | 52.3 | 5.15 (m) |
| *m*-Tyr-A3 | 38.3, $CH_2$ | 2.69 (m)/2.95 (m) | *m*-Tyr-AH2 | overlap | 40.3 | 2.86 (m)/2.92 (m) |
| *m*-Tyr-A4 | 140.1, C | | | | 139.1 | |
| *m*-Tyr-A5 | 114.9, CH[a] | 6.64 (m) | overlap | overlap | 115.1 | 6.64 (m) |
| *m*-Tyr-A6 | 158.6, C-O[a] | | | | 158.8 | |
| *m*-Tyr-A7 | 117.0, CH[a] | 6.69 (m) | overlap | overlap | 117.3 | 6.64 (m) |
| *m*-Tyr-A8 | 130.7, CH[a] | 7.09 (m) | overlap | overlap | 130.8 | 7.09 (m) |
| *m*-Tyr-A9 | 121.3, CH[a] | 6.72 (m) | overlap | overlap | 121.6 | 6.72 (m) |
| *m*-Tyr-A11 | 173.1, N-C=O | | | | 172.8 | |
| *m*-Tyr-A12 | 22.3, $CH_3$ | 1.87 (s) | None | *m*-Tyr-AC11 | 22.6 | 1.96 (s) |
| *m*-Tyr-B1 | 176.4, COOH | | | | 176.5 | |
| *m*-Tyr-B2 | 55.9, CH | 4.50 (m) | *m*-Tyr-BH3 | *m*-Tyr-BC1, C3, C4; Ureido | 55.9 | 4.50 (m) |
| *m*-Tyr-B3 | 39.1, $CH_2$ | 2.92 (m)/2.97 (m) | *m*-Tyr-BH2 | | 39.1 | 2.92 (m)/2.97 (m) |
| *m*-Tyr-B4 | 139.8, C | | | | 139.8 | |
| *m*-Tyr-B5 | 114.7, CH[a] | 6.64 (m) | overlap | overlap | 114.7 | 6.64 (m) |
| *m*-Tyr-B6 | 158.4, C-O[a] | | | | 158.4 | |
| *m*-Tyr-B7 | 117.4, CH[a] | 6.64 (m) | overlap | overlap | 117.4 | 6.69 (m) |
| *m*-Tyr-B8 | 130.4, CH[a] | 7.05 (m) | overlap | overlap | 130.4 | 7.05 (m) |
| *m*-Tyr-B9 | 121.9, CH[a] | 6.66 (m) | overlap | overlap | 121.8 | 6.66 (m) |

[a], $^{13}$C NMR data were assigned based on combination of the HMBC correlations and the comparison with that of *N*-acetylmureidomycin E and G. Abbreviations for the structure units: *m*-Tyr (*m*-tyrosine), AMBA (2-amino-3-methylaminobutyric acid).

Table S5 NMR data for component (**4**) in $CD_3OD$.

| Position | *N*-acetylmyreidomycin H1 | | | | iso-*N*-acetylmyreidomycin H1 (**4$_{iso}$**) | |
|---|---|---|---|---|---|---|
| | δ$_C$-ppm | δ$_H$-ppm (*J*, Hz) | COSY | Key HMBC | δ$_C$-ppm | δ$_H$-ppm (*J*, Hz) |
| Uracil-2 | 152.1, N-C=O | | | | 152.1 | |
| Uracil-4 | 165.9, N-C=O | | | | 166.1 | |
| Uracil-5 | 103.5, CH | 5.59 (d, 8.5) | Uracil-H6 | Uracil-C4, C6 | 103.4 | 5.76 (d, 8.0) |
| Uracil-6 | 141.2, CH | 7.14 (d, 8.5) | Uracil-H5 | Uracil-C2, C4, C5; Sugar-C1 | 142.0 | 7.42 (d, 8.0) |
| Sugar-1 | 94.5, O-CH-N | 6.04 (d, 2.5) | Sugar-H2 | Sugar-C2, C3 (w), C4; Uracil-C2, C6 | 95.2 | 6.02 (d, 2.5) |
| Sugar-2 | 73.8, O-CH | 4.38 (m) | Sugar-H1, H3 | Sugar-C4 (w) | 74.1 | 4.50 (m) |
| Sugar-3 | 35.0, CH2 | 2.60 (m)/2.93 (m) | Sugar-H2, H5 | Sugar-C1, C2, C4, C5 | 35.0 | 2.60 (m) / 2.93 (m) |
| Sugar-4 | 143.6, >C= | | | | 144.2 | |
| Sugar-5 | 98.3, -CH= | 5.98 (s) | Sugar-H3 | Sugar-C3, C4; AMBA-C1 | 98.0 | 5.93 (s) |
| AMBA-1 | 168.3, C=O | | | | 168.1 | |
| AMBA-2 | 56.5, CH | 4.71 (d, 8.5) | AMBA-H3 | AMBA-C1, C3, C4; Leu-C1 | 57.1 | 4.69 (d, 8.5) |
| AMBA-3 | 52.5, CH | 4.90 (m) | AMBA-H2, H4 | None | 55.8 | 4.18 (m) |
| AMBA-4 | 14.0, $CH_3$ | 1.19 (d, 6.5) | AMBA-H3 | AMBA-C2, C3 | 14.5 | 0.74 (d, 6.5) |
| AMBA-6 | 31.2, N-$CH_3$ | 3.01 (s) | None | AMBA-C3; *m*-Tyr-AC1 | 28.7 | 2.66 (s) |
| Leu-1 | 176.1, N-C=O | | | | 175.9 | |
| Leu-2 | 53.7, CH | 4.22 (m) | Leu-H3 | Leu-C1, C3, C4 (w); Ureido | 53.7 | 4.22 (m) |
| Leu-3 | 42.4, $CH_2$ | 1.45 (m) | Leu-H2, H4 | Leu-C1, C2, C4, C5, C6 | 42.3 | 1.45 (m) |
| Leu-4 | 25.9, CH | 1.64 (m) | Leu-H3, H5, H6 | Leu-C2, C3, C5, C6 | 25.9 | 1.64 (m) |
| Leu-5 | 23.5, $CH_3$ | 0.90 (d, 6.5) | Leu-H4 | Leu-C3, C4, C6 | 23.5 | 0.90 (d, 6.5) |
| Leu-6 | 22.1, $CH_3$ | 0.91 (d, 7.0) | Leu-H4 | Leu-C3, C4, C5 | 22.1 | 0.91 (d, 7.0) |
| Ureido | 159.7, N-C=O | | | | 159.7 | |
| *m*-Tyr-A1 | 174.2, N-C=O | | | | 173.5 | |
| *m*-Tyr-A2 | 52.5, CH | 4.94 (dd, 4.0, 10.0) | *m*-Tyr-AH3 | *m*-Tyr-AC1, C3, C4 | 52.3 | 5.16 (m) |
| *m*-Tyr-A3 | 38.2, $CH_2$ | 2.69 (m) / 2.95 (m) | *m*-Tyr-AH2 | overlap | 40.3 | 2.86 (m) / 2.92 (m) |
| *m*-Tyr-A4 | 140.1, C | | | | 139.1 | |
| *m*-Tyr-A5 | 114.9, CH[a] | 6.65 (m) | overlap | overlap | 115.1 | 6.65 (m) |
| *m*-Tyr-A6 | 158.6, C-O[a] | | | | 158.8 | |
| *m*-Tyr-A7 | 117.0, CH[a] | 6.68 (m) | overlap | overlap | 117.3 | 6.62 (d, 8.5) |
| *m*-Tyr-A8 | 130.7, CH[a] | 7.10 (m) | overlap | overlap | 130.8 | 7.10 (m) |
| *m*-Tyr-A9 | 121.3, CH[a] | 6.71 (d, 7.5) | overlap | overlap | 121.6 | 6.67 (m) |
| *m*-Tyr-A11 | 173.1, N-C=O | | | | 172.7 | |
| *m*-Tyr-A12 | 22.3, $CH_3$ | 1.87 (s) | None | *m*-Tyr-AC11 | 22.6 | 1.96 (s) |
| *m*-Tyr-B1 | 176.2, COOH | | | | 176.2 | |
| *m*-Tyr-B2 | 55.9, CH | 4.47 (m) | *m*-Tyr-BH3 | *m*-Tyr-BC1, C3, C4; Ureido | 55.9 | 4.47 (m) |
| *m*-Tyr-B3 | 39.1, $CH_2$ | 2.92 (m) | *m*-Tyr-BH2 | | 39.2 | 2.93 (m) |
| *m*-Tyr-B4 | 139.8, C | | | | 139.8 | |
| *m*-Tyr-B5 | 114.7, CH[a] | 6.64 (m) | overlap | overlap | 114.7 | 6.64 (m) |
| *m*-Tyr-B6 | 158.4, C-O[a] | | | | 158.4 | |
| *m*-Tyr-B7 | 117.4, CH[a] | 6.66 (m) | overlap | overlap | 117.4 | 6.66 (m) |
| *m*-Tyr-B8 | 130.4, CH[a] | 7.05 (dd, 7.5, 7.5) | overlap | overlap | 130.4 | 7.05 (dd, 7.5, 7.5) |
| *m*-Tyr-B9 | 121.9, CH[a] | 6.66 (m) | overlap | overlap | 121.8 | 6.66 (m) |

[a], $^{13}C$ NMR data were assigned based on combination of the HMBC correlations and the comparison with that of *N*-acetylmureidomycin E and G. Abbreviations for the structure units: *m*-Tyr (*m*-tyrosine), AMBA (2-amino-3-methylaminobutyric acid).

Table S6 NMR data for component (**5**) in $CD_3OD$.

| Position | *N*-acetylmyreidomycin G | | | | iso-*N*-acetylmyreidomycin G (**5iso**) | |
|---|---|---|---|---|---|---|
| | $\delta_C$-ppm | $\delta_H$-ppm (*J*, Hz) | COSY | Key HMBC | $\delta_C$-ppm | $\delta_H$-ppm (*J*, Hz) |
| Uracil-2 | 152.1, N-C=O | | | | 152.1 | |
| Uracil-4 | 165.9, N-C=O | | | | 166.1 | |
| Uracil-5 | 103.5, CH | 5.60 (d, 8.0) | Uracil-H6 | Uracil-C4, C6 | 103.4 | 5.76 (d, 8.0) |
| Uracil-6 | 141.3, CH | 7.15 (d, 8.0) | Uracil-H5 | Uracil-C2, C4, C5; Sugar-C1 | 142.0 | 7.41 (d, 8.5) |
| Sugar-1 | 94.5, O-CH-N | 6.05 (d, 3.0) | Sugar-H2 | Sugar-C2, C3 (w), C4; Uracil-C2, C6 | 95.2 | 6.03 (d, 2.5) |
| Sugar-2 | 73.9, O-CH | 4.38 (m) | Sugar-H1, H3 | Sugar-C4 (w) | 74.1 | 4.48 (m) |
| Sugar-3 | 34.9, $CH_2$ | 2.59 (m)/2.89 (m) | Sugar-H2, H5 | Sugar-C1, C2, C4, C5 | 35.0 | 2.61 (m)/2.92 (m) |
| Sugar-4 | 143.6, >C= | | | | 144.1 | |
| Sugar-5 | 98.3, -CH= | 5.98 (s) | Sugar-H3 | Sugar-C3 (w), C4; AMBA-C1 | 98.1 | 5.93 (s) |
| AMBA-1 | 168.2, C=O | | | | 168.0 | |
| AMBA-2 | 56.7, CH | 4.69 (d, 8.5) | AMBA-H3 | AMBA-C1, C3, C4; Met-C1 | 57.2 | 4.68 (d, 6.5) |
| AMBA-3 | 52.5, CH | 4.89 (m) | AMBA-H2, H4 | None | 55.6 | 4.17 (m) |
| AMBA-4 | 14.0, $CH_3$ | 1.19 (d, 7.0) | AMBA-H3 | AMBA-C2, C3 | 14.4 | 0.72 (d, 6.5) |
| AMBA-6 | 31.3, N-$CH_3$ | 3.01 (s) | None | AMBA-C3; *m*-Tyr-AC1 | 28.7 | 2.64 (s) |
| Met-1 | 175.0, N-C=O | | | | 174.7 | |
| Met-2 | 54.3, CH | 4.32 (dd, 5.0, 8.0) | Met-H3 | Met-C1, C3, C4; Ureido | 54.3 | 4.28 (dd, 5.5, 8.5) |
| Met-3 | 33.5, $CH_2$ | 1.82 (m)/1.96 (m) | Met-H2, H4 | Met-C1, C2, C4 | 33.4 | 1.82 (m)/1.96 (m) |
| Met-4 | 31.1, S-$CH_2$ | 2.47 (m) | Met-H3 | Met-C2, C3, C6 | 31.1 | 2.47 (m) |
| Met-6 | 15.4, $CH_3$ | 2.04 (s) | None | Met-C4 | 15.4 | 2.03 (s) |
| Ureido | 159.7, N-C=O | | | | 159.7 | |
| *m*-Tyr-A1 | 174.2, N-C=O | | | | 173.6 | |
| *m*-Tyr-A2 | 52.5, CH | 4.93 (dd, 4.0, 10.0) | *m*-Tyr-AH3 | *m*-Tyr-AC1, C3, C4 | 52.3 | 5.15 (m) |
| *m*-Tyr-A3 | 38.3, $CH_2$ | 2.68 (m)/2.91 (m) | *m*-Tyr-AH2 | *m*-Tyr-AC1, C2, C4, C5, C9 | 40.3 | 2.86 (m) |
| *m*-Tyr-A4 | 140.1, C | | | | 139.1 | |
| *m*-Tyr-A5 | 114.9, CH[a] | 6.65 (m) | overlap | *m*-Tyr-AC6, C7 | 115.1 | 6.65 (m) |
| *m*-Tyr-A6 | 158.7, C-O[a] | | | | 158.9 | |
| *m*-Tyr-A7 | 117.0, CH[a] | 6.68 (m) | *m*-Tyr-AH8 | *m*-Tyr-AC5 | 117.4 | 6.61 (m) |
| *m*-Tyr-A8 | 130.7, CH[a] | 7.10 (m) | *m*-Tyr-AH7, H9 | *m*-Tyr-AC4, C6 | 130.8 | 7.10 (m) |
| *m*-Tyr-A9 | 121.3, CH[a] | 6.72 (d, 7.5) | *m*-Tyr-AH8 | *m*-Tyr-AC5, C7 | 121.6 | 6.65 (d, 7.5) |
| *m*-Tyr-A11 | 173.1, N-C=O | | | | 172.7 | |
| *m*-Tyr-A12 | 22.3, $CH_3$ | 1.87 (s) | None | *m*-Tyr-AC11 | 22.6 | 1.96 (s) |
| Phe-1 | 176.4, COOH | | | | 176.4 | |
| Phe-2 | 56.0, CH | 4.48 (m) | Phe-H3 | Phe-C1, C3, C4; Ureido | 56.0 | 4.48 (m) |
| Phe-3 | 39.2, $CH_2$ | 2.98 (m) | Phe-H2 | Phe-C1, C2, C4, C5, C9 | 39.3 | 3.08 (m) |
| Phe-4 | 138.5, C | | | | 138.6 | |
| Phe-5 | 130.6, CH[a] | 7.18 (m) | overlap | Phe-C3, C7, C9 | 130.6 | 7.18 (m) |
| Phe-6 | 129.4, CH[a] | 7.23 (m) | overlap | Phe-C4, C8 | 129.4 | 7.23 (m) |
| Phe-7 | 127.7, CH[a] | 7.17 (m) | overlap | Phe-C5, C9 | 127.4 | 7.17 (m) |
| Phe-8 | 129.4, CH[a] | 7.23 (m) | overlap | Phe-C4, C6 | 129.4 | 7.23 (m) |
| Phe-9 | 130.6, CH[a] | 7.18 (m) | overlap | Phe-C3, C5, C7 | 130.6 | 7.18 (m) |

[a], $^{13}C$ NMR data were assigned based on combination of the HMBC correlations and the comparison with that of *N*-acetylmureidomycin E. Abbreviations for the structure units: *m*-Tyr (*m*-tyrosine), AMBA (2-amino-3-methylaminobutyric acid).

Table S7 NMR data for component (**3’**) in $CD_3OD$.

| Position | *N*-acetylmyreidomycin B | | | | iso-*N*-acetylmyreidomycin B (**3’$_{iso}$**) | |
|---|---|---|---|---|---|---|
| | $\delta_C$-ppm | $\delta_H$-ppm (*J*, Hz) | COSY | Key HMBC | $\delta_C$-ppm | $\delta_H$-ppm (*J*, Hz) |
| Uracil-2 | 155.1, N-C=O | | | | 155.1 | |
| Uracil-4 | 172.5, N-C=O | | | | 172.7 | |
| Uracil-5 | 31.8, $CH_2$ | 2.57 (m)/2.65 (m) | Uracil-H6 | Uracil-C4, C6 | 32.0 | 2.57 (m)/2.65 (m) |
| Uracil-6 | 37.7, $CH_2$ | 3.08 (m) | Uracil-H5 | Uracil-C2, C4, C5; Sugar-C1 | 38.3 | 2.92 (m) |
| Sugar-1 | 93.4, O-CH-N | 6.04 (d, 4.5) | Sugar-H2 | Sugar-C2, C3 (w), C4; Uracil-C2, C6 | 94.1 | 6.04 (d, 4.5) |
| Sugar-2 | 71.5, O-CH | 4.22 (m) | Sugar-H1, H3 | Sugar-C4 | 72.1 | 4.42 (m) |
| Sugar-3 | 35.9, $CH_2$ | 2.61 (m)/2.92 (m) | Sugar-H2, H5 | Sugar-C1, C2, C4, C5 | 36.0 | 2.61 (m)/2.92 (m) |
| Sugar-4 | 143.1, >C= | | | | 144.0 | |
| Sugar-5 | 97.1, -CH= | 5.88 (s) | Sugar-H3 | Sugar-C3, C4; AMBA-C1 | 96.8 | 5.80 (s) |
| AMBA-1 | 168.1, C=O | | H6b, H8 | | 167.8 | |
| AMBA-2 | 56.6, CH | 4.65 (d, 9.0) | AMBA-H3 | AMBA-C1, C3, C4; Met-C1 | 57.2 | 4.69 (d, 7.5) |
| AMBA-3 | 52.2, CH | 4.93 (m) | AMBA-H2, H4 | None | 55.6 | 4.17 (m) |
| AMBA-4 | 14.2, $CH_3$ | 1.21 (d, 6.5) | AMBA-H3 | AMBA-C2, C3 | 14.0 | 0.76 (d, 6.5) |
| AMBA-6 | 31.0, N-$CH_3$ | 3.05 (s) | None | AMBA-C3; *m*-Tyr-AC1 | 28.8 | 2.67 (s) |
| Met-1 | 175.0, N-C=O | | | | 174.8 | |
| Met-2 | 54.2, CH | 4.36 (m) | Met-H3 | Met-C1, C3, C4; Ureido | 54.2 | 4.31 (m) |
| Met-3 | 33.6, $CH_2$ | 1.82 (m)/1.98 (m) | Met-H2, H4 | Met-C1, C2, C4 | 33.5 | 1.82 (m)/1.98 (m) |
| Met-4 | 31.1, S-$CH_2$ | 2.50 (m) | Met-H3 | Met-C2, C3, C6 | 30.9 | 2.50 (m) |
| Met-6 | 15.4, $CH_3$ | 2.04 (s) | None | Met-C4 | 14.6 | 2.04 (s) |
| Ureido | 159.6, N-C=O | | | | 159.6 | |
| *m*-Tyr-A1 | 174.2, N-C=O | | | | 173.5 | |
| *m*-Tyr-A2 | 52.2, CH | 4.96 (dd, 3.5, 10.5) | *m*-Tyr-AH3 | *m*-Tyr-AC1, C3, C4 | 52.2 | 5.16 (m) |
| *m*-Tyr-A3 | 38.1, $CH_2$ | 2.69 (m) / 2.92 (m) | *m*-Tyr-AH2 | overlap | 40.3 | 2.87 (m) |
| *m*-Tyr-A4 | 140.2, C | | | | 139.1 | |
| *m*-Tyr-A5 | 114.8, CH[a] | 6.67 (m) | overlap | overlap | 115.0 | 6.67 (m) |
| *m*-Tyr-A6 | 158.7, C-O[a] | | | | 158.8 | |
| *m*-Tyr-A7 | 116.8, CH[a] | 6.67 (m) | overlap | overlap | 117.4 | 6.67 (m) |
| *m*-Tyr-A8 | 130.7, CH[a] | 7.11 (m) | overlap | overlap | 130.7 | 7.11 (m) |
| *m*-Tyr-A9 | 121.1, CH[a] | 6.70 (m) | overlap | overlap | 121.6 | 6.67 (m) |
| *m*-Tyr-A11 | 173.2, N-C=O | | | | 172.7 | |
| *m*-Tyr-A12 | 22.3, $CH_3$ | 1.86 (s) | None | *m*-Tyr-AC11 | 22.6 | 1.98 (s) |
| *m*-Tyr-B1 | 176.0, COOH | | | | 176.0 | |
| *m*-Tyr-B2 | 55.6, CH | 4.51 (m) | *m*-Tyr-BH3 | *m*-Tyr-BC1, C3, C4; Ureido | 55.6 | 4.51 (m) |
| *m*-Tyr-B3 | 39.1, $CH_2$ | 2.93 (m)/2.99 (m) | *m*-Tyr-BH2 | | 39.1 | 2.93 (m)/2.99 (m) |
| *m*-Tyr-B4 | 139.7, C | | | | 139.7 | |
| *m*-Tyr-B5 | 114.8, CH[a] | 6.67 (m) | overlap | overlap | 114.8 | 6.67 (m) |
| *m*-Tyr-B6 | 158.4, C-O[a] | | | | 158.4 | |
| *m*-Tyr-B7 | 117.4, CH[a] | 6.67 (m) | overlap | overlap | 117.4 | 6.67 (m) |
| *m*-Tyr-B8 | 130.4, CH[a] | 7.06 (m) | overlap | overlap | 130.4 | 7.06 (m) |
| *m*-Tyr-B9 | 121.8, CH[a] | 6.67 (m) | overlap | overlap | 121.8 | 6.67 (m) |

[a], $^{13}C$ NMR data were assigned based on combination of the HMBC correlations and the comparison with that of *N*-acetylmureidomycin E and G. Abbreviations for the structure units: *m*-Tyr (*m*-tyrosine), AMBA (2-amino-3-methylaminobutyric acid).

Table S8 NMR data for component (**4'**) in $CD_3OD$.

| Position | *N*-acetylmyreidomycin H2 | | | | iso-*N*-acetylmyreidomycin H2 (**4'$_{iso}$**) | |
|---|---|---|---|---|---|---|
| | δC-ppm | δH-ppm (*J*, Hz) | COSY | Key HMBC | δC-ppm | δH-ppm (*J*, Hz) |
| Uracil-2 | 155.2, N-C=O | | | | 155.1 | |
| Uracil-4 | 172.5, N-C=O | | | | 172.6 | |
| Uracil-5 | 31.8, $CH_2$ | 2.51 (m)/2.65 (m) | Uracil-H6 | Uracil-C4, C6 | 32.0 | 2.51 (m)/2.65 (m) |
| Uracil-6 | 37.7, $CH_2$ | 3.05 (m) | Uracil-H5 | Uracil-C2, C4, C5; Sugar-C1 | 38.4 | 2.89 (m) |
| Sugar-1 | 93.4, O-CH-N | 6.03 (d, 4.5) | Sugar-H2 | Sugar-C2, C3, C4; Uracil-C2, C6 | 94.2 | 6.03 (d, 4.5) |
| Sugar-2 | 71.4, O-CH | 4.22 (m) | Sugar-H1, H3 | Sugar-C4 (w) | 72.1 | 4.48 (m) |
| Sugar-3 | 35.9, $CH_2$ | 2.60 (m)/2.90 (m) | Sugar-H2, H5 | Sugar-C1, C2, C4, C5 | 36.1 | 2.60 (m)/2.90 (m) |
| Sugar-4 | 143.2, >C= | | | | 144.0 | |
| Sugar-5 | 97.0, -CH= | 5.87 (s) | Sugar-H3 | Sugar-C3, C4; AMBA-C1 | 96.8 | 5.80 (s) |
| AMBA-1 | 168.1, C=O | | | | 167.8 | |
| AMBA-2 | 56.3, CH | 4.68 (d, 8.5) | AMBA-H3 | AMBA-C1, C3, C4; Leu-C1 | 57.0 | 4.68 (d, 7.0) |
| AMBA-3 | 52.3, CH | 4.91 (m) | AMBA-H2, H4 | None | 55.8 | 4.18 (m) |
| AMBA-4 | 14.1, $CH_3$ | 1.18 (d, 6.5) | AMBA-H3 | AMBA-C2, C3 | 14.5 | 0.76 (d, 6.5) |
| AMBA-6 | 30.9, N-$CH_3$ | 3.04 (s) | None | AMBA-C3; *m*-Tyr-AC1 | 28.7 | 2.67 (s) |
| Leu-1 | 176.0, N-C=O | | | | 175.8 | |
| Leu-2 | 53.7, CH | 4.22 (m) | Leu-H3 | Leu-C1, C3, C4 (w); Ureido | 53.7 | 4.22 (m) |
| Leu-3 | 42.4, $CH_2$ | 1.48 (m) | Leu-H2, H4 | Leu-C1, C2, C4, C5, C6 | 42.3 | 1.48 (m) |
| Leu-4 | 25.9, CH | 1.66 (m) | Leu-H3, H5, H6 | Leu-C2, C3, C5, C6 | 25.9 | 1.66 (m) |
| Leu-5 | 23.6, $CH_3$ | 0.90 (d, 8.0) | Leu-H4 | Leu-C3, C4, C6 | 23.5 | 0.90 (d, 8.0) |
| Leu-6 | 22.1, $CH_3$ | 0.92 (d, 8.0) | Leu-H4 | Leu-C3, C4, C5 | 22.1 | 0.92 (d, 8.0) |
| Ureido | 159.7, N-C=O | | | | 159.7 | |
| *m*-Tyr-A1 | 174.2, N-C=O | | | | 173.5 | |
| *m*-Tyr-A2 | 52.3, CH | 4.95 (dd, 4.0, 10.0) | *m*-Tyr-AH3 | *m*-Tyr-AC1, C3, C4 | 52.2 | 5.17 (m) |
| *m*-Tyr-A3 | 38.1, $CH_2$ | 2.65 (m)/2.89 (m) | *m*-Tyr-AH2 | overlap | 40.4 | 2.86 (m) |
| *m*-Tyr-A4 | 140.2, C | | | | 139.2 | |
| *m*-Tyr-A5 | 114.8, CH[a] | 6.66 (m) | overlap | overlap | 115.1 | 6.66 (m) |
| *m*-Tyr-A6 | 158.7, C-O[a] | | | | 158.8 | |
| *m*-Tyr-A7 | 116.7, CH[a] | 6.68 (m) | overlap | overlap | 117.3 | 6.62 (m) |
| *m*-Tyr-A8 | 130.7, CH[a] | 7.11 (m) | overlap | overlap | 130.7 | 7.11 (m) |
| *m*-Tyr-A9 | 121.1, CH[a] | 6.70 (d, 7.5) | overlap | overlap | 121.6 | 6.67 (m) |
| *m*-Tyr-A11 | 173.1, N-C=O | | | | 172.7 | |
| *m*-Tyr-A12 | 22.3, $CH_3$ | 1.87 (s) | None | *m*-Tyr-AC11 | 22.6 | 1.97 (s) |
| *m*-Tyr-B1 | 176.1, COOH | | | | 176.1 | |
| *m*-Tyr-B2 | 55.8, CH | 4.48 (m) | *m*-Tyr-BH3 | *m*-Tyr-BC1, C3, C4; Ureido | 55.8 | 4.48 (m) |
| *m*-Tyr-B3 | 39.1, $CH_2$ | 2.91 (m)/2.99 (m) | *m*-Tyr-BH2 | | 39.1 | 2.91 (m)/2.99 (m) |
| *m*-Tyr-B4 | 139.8, C | | | | 139.8 | |
| *m*-Tyr-B5 | 114.7, CH[a] | 6.65 (m) | overlap | overlap | 114.7 | 6.65 (m) |
| *m*-Tyr-B6 | 158.4, C-O[a] | | | | 158.4 | |
| *m*-Tyr-B7 | 117.4, CH[a] | 6.67 (m) | overlap | overlap | 117.5 | 6.67 (m) |
| *m*-Tyr-B8 | 130.4, CH[a] | 7.06 (dd, 8.0, 8.0) | overlap | overlap | 130.4 | 7.06 (dd, 8.0, 8.0) |
| *m*-Tyr-B9 | 121.9, CH[a] | 6.67 (m) | overlap | overlap | 121.8 | 6.67 (m) |

[a], $^{13}C$ NMR data were assigned based on the combination of HMBC correlations and the comparison with that of *N*-acetylmureidomycin E and G. Abbreviations for the structure units: *m*-Tyr (*m*-tyrosine), AMBA (2-amino-3-methylaminobutyric acid).

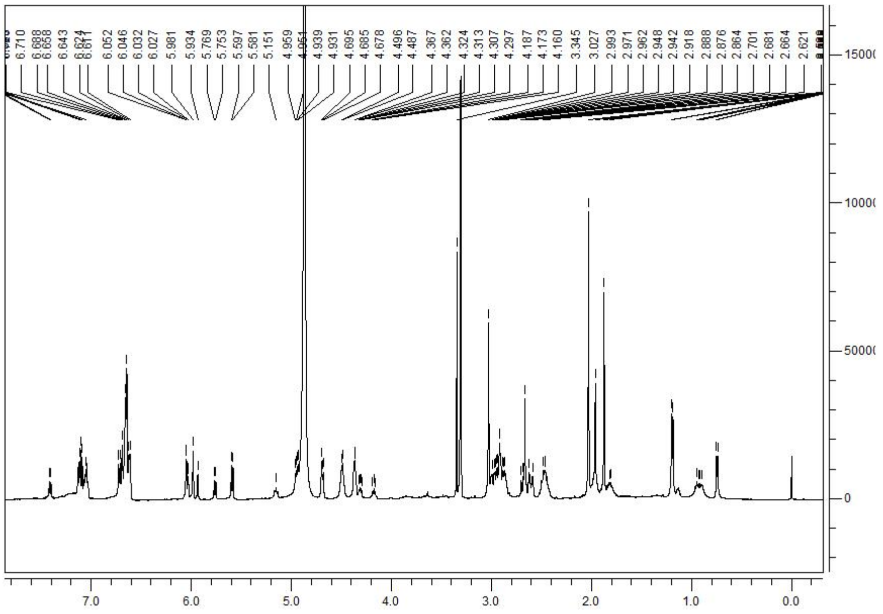


Figure S7. $^{1}$H NMR spectrum (500 MHz) of component **(3)** in $CD_3OD$.

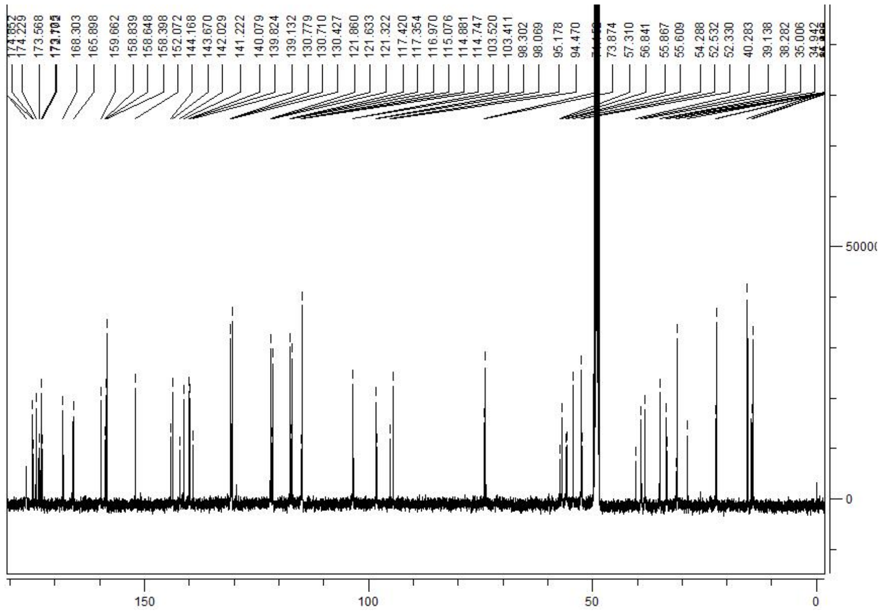


Figure S8. $^{13}$C NMR spectrum (125 MHz) of component **(3)** in $CD_3OD$.

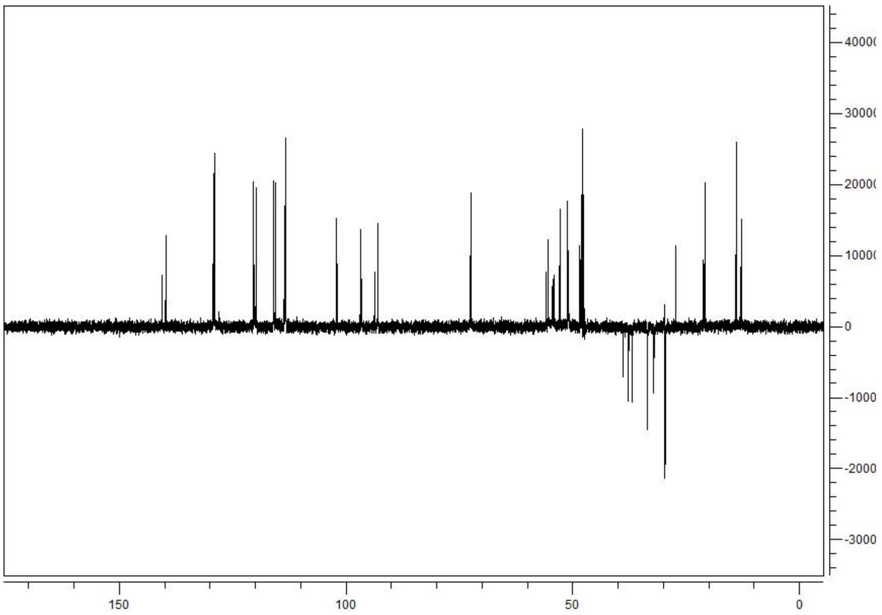


Figure S9. Dept135 spectrum of component **(3)** in $CD_3OD$.

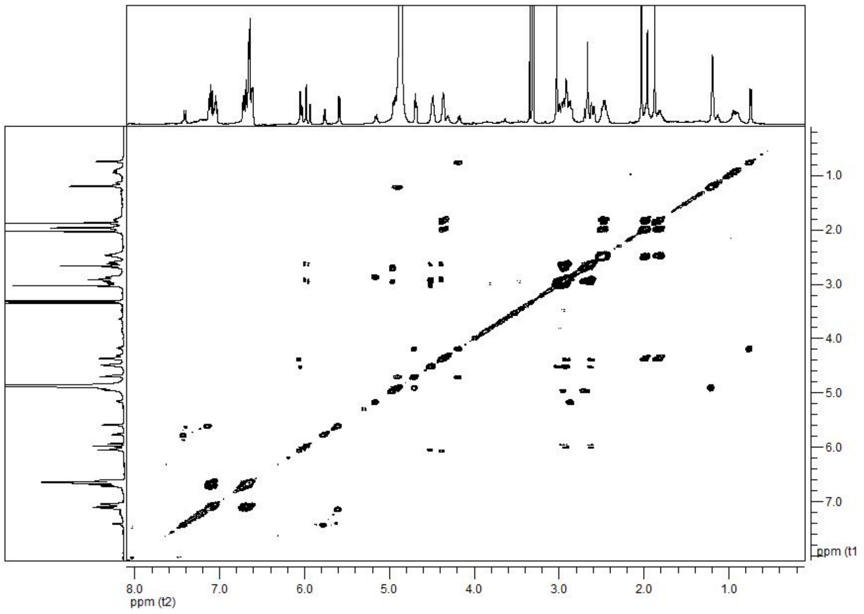


Figure S10. COSY spectrum of component **(3)** in $CD_3OD$.

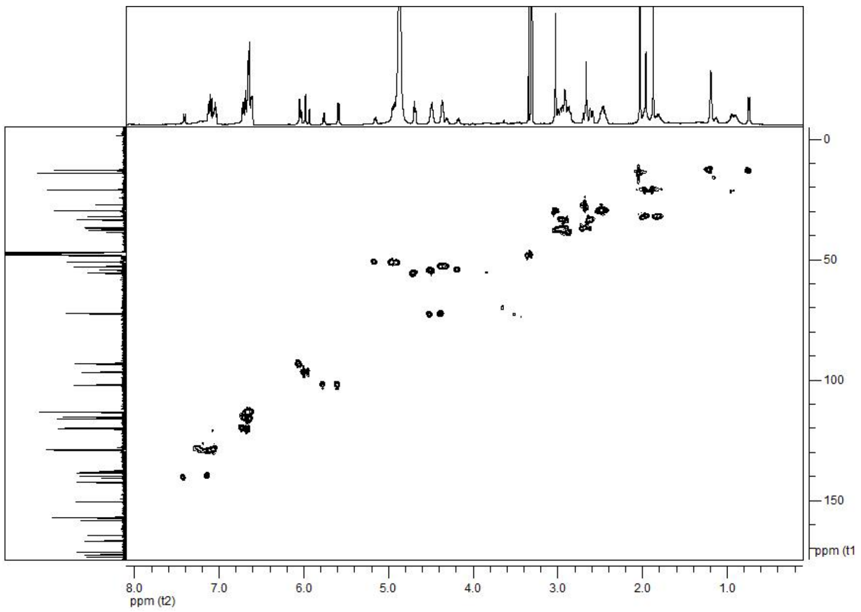


Figure S11. HSQC spectrum of component **(3)** in $CD_3OD$.

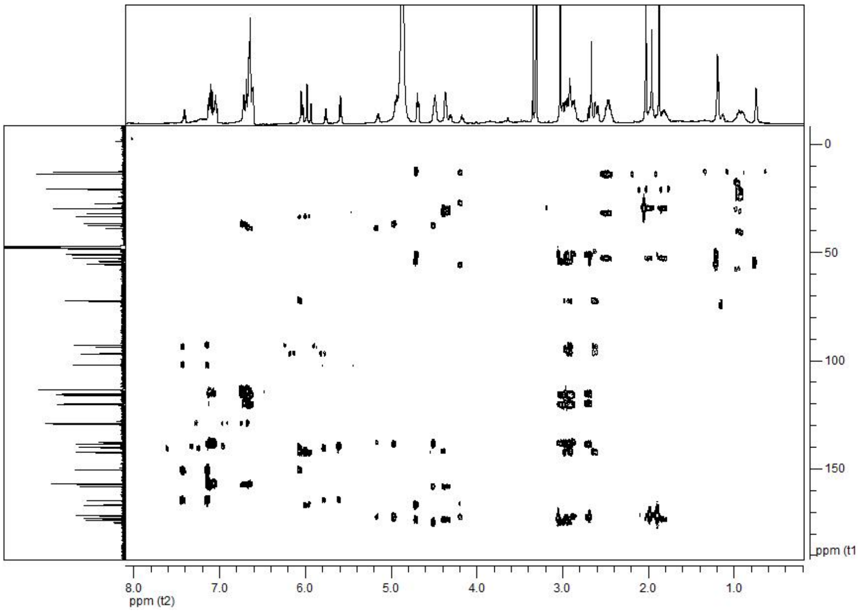


Figure S12. HMBC spectrum of component **(3)** in $CD_3OD$.

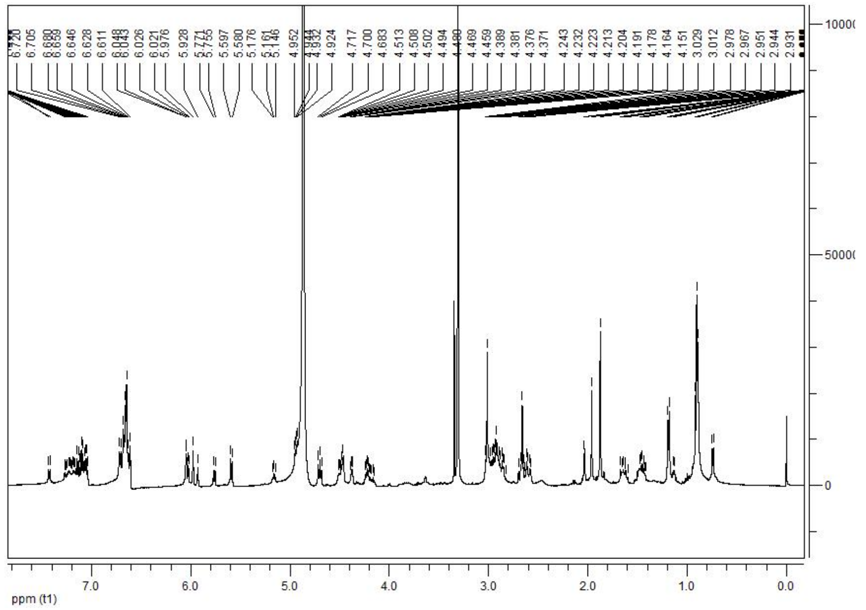

Figure S13. $^{1}H$ NMR spectrum (500 MHz) of component **(4)** in $CD_3OD$.

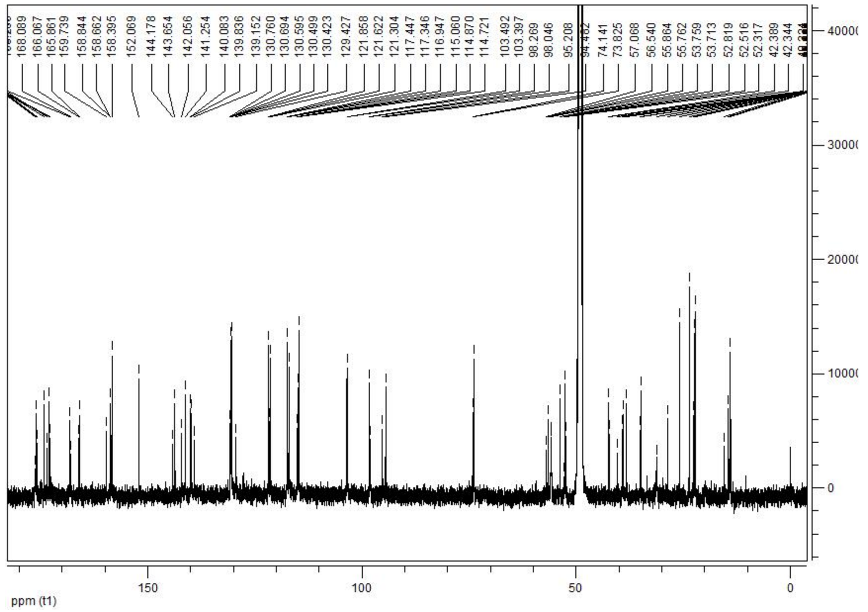

Figure S14. $^{13}C$ NMR spectrum (125 MHz) of component **(4)** in $CD_3OD$.

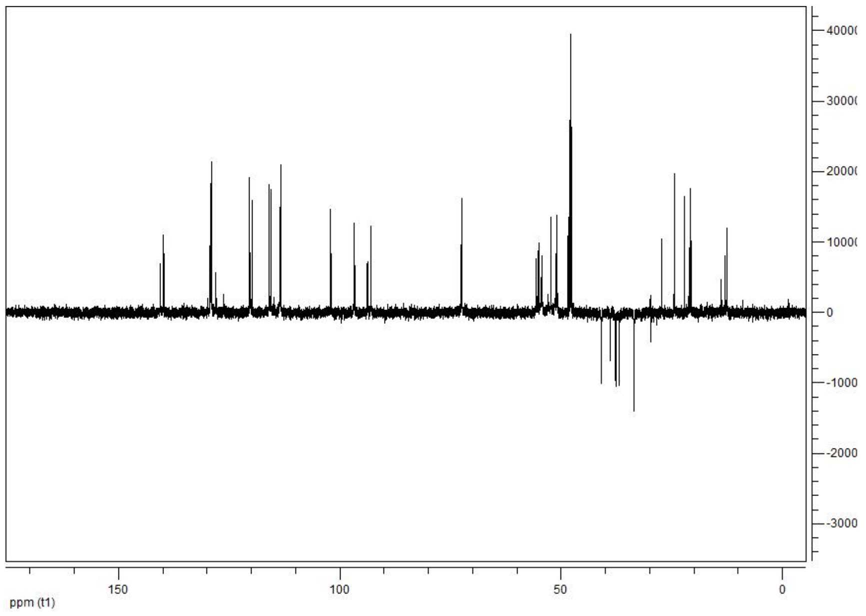


Figure S15. Dept135 spectrum of component **(4)** in $CD_3OD$.

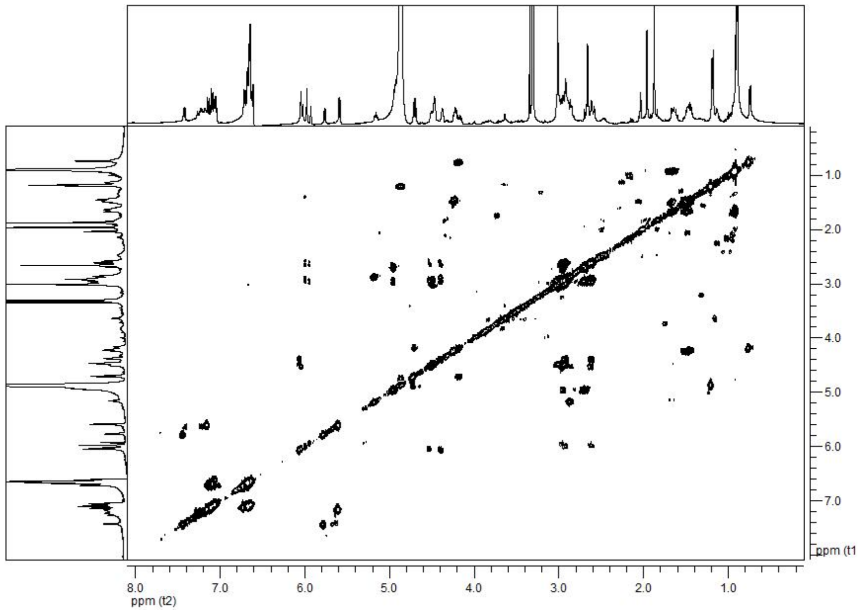


Figure S16. COSY spectrum of component **(4)** in $CD_3OD$.

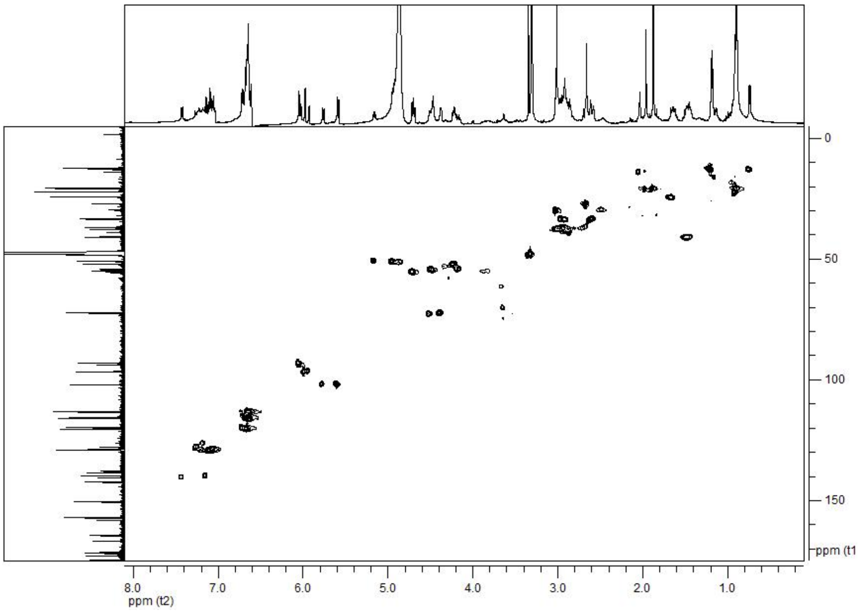


Figure S17. HSQC spectrum of component **(4)** in $CD_3OD$.

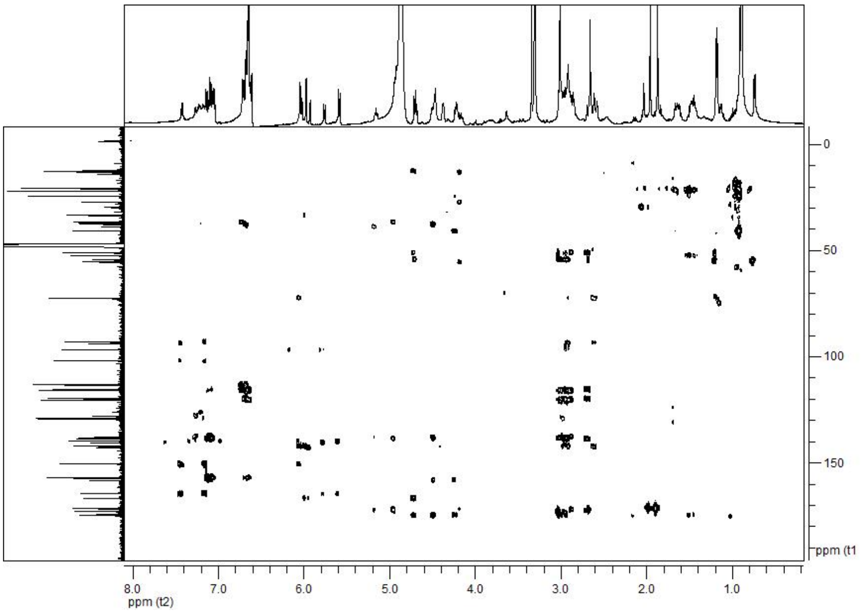


Figure S18. HMBC spectrum of component **(4)** in $CD_3OD$.

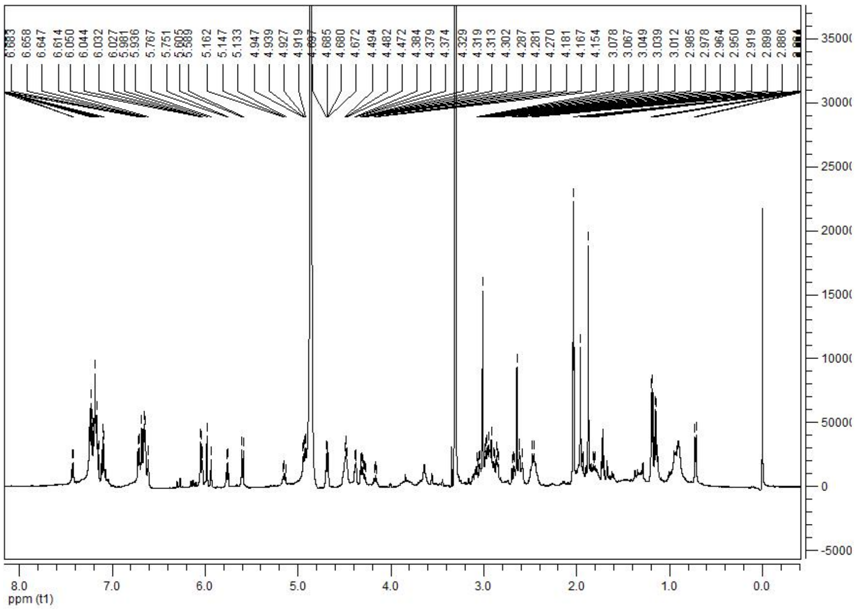


Figure S19. $^{1}$H NMR spectrum (500 MHz) of component **(5)** in $CD_3OD$.

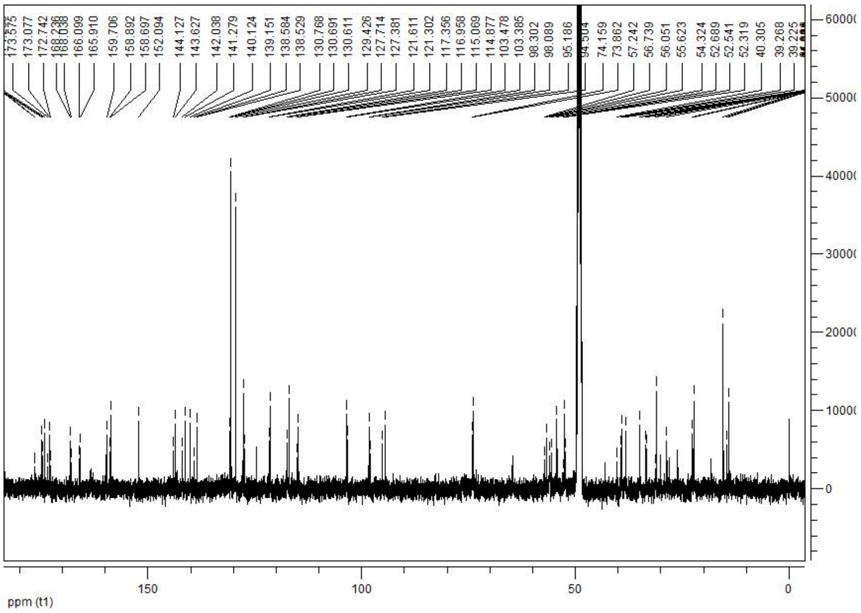


Figure S20. $^{13}$C NMR spectrum (125 MHz) of component **(5)** in $CD_3OD$.

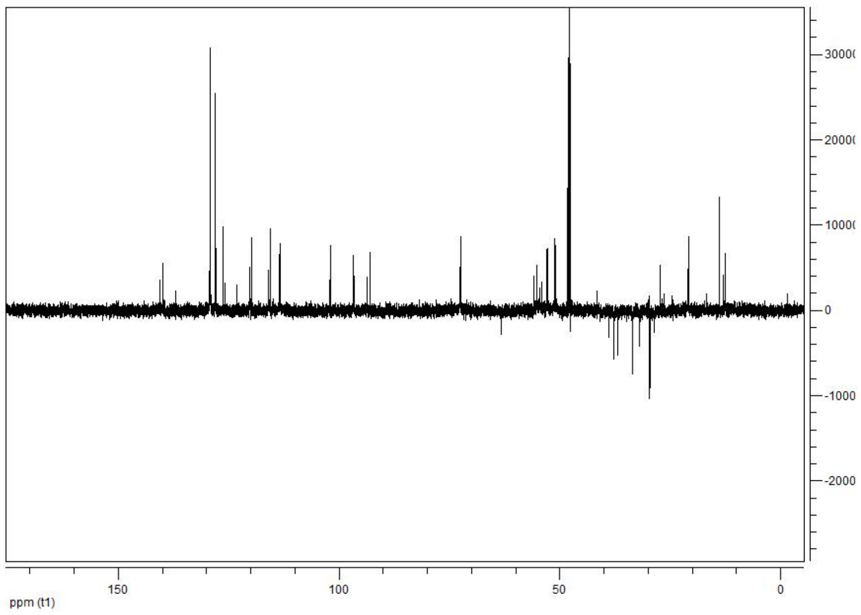


Figure S21. Dept135 spectrum of component **(5)** in $CD_3OD$.

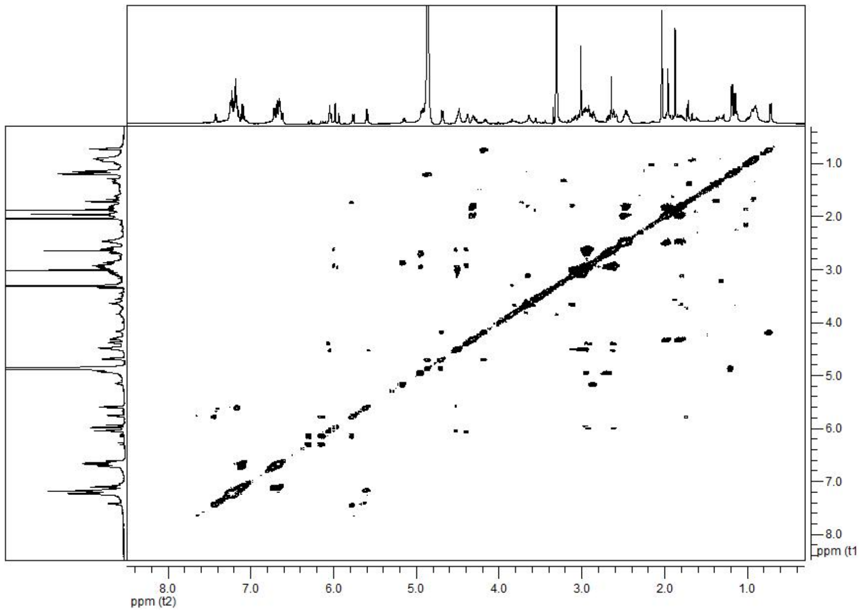


Figure S22. COSY spectrum of component **(5)** in $CD_3OD$.

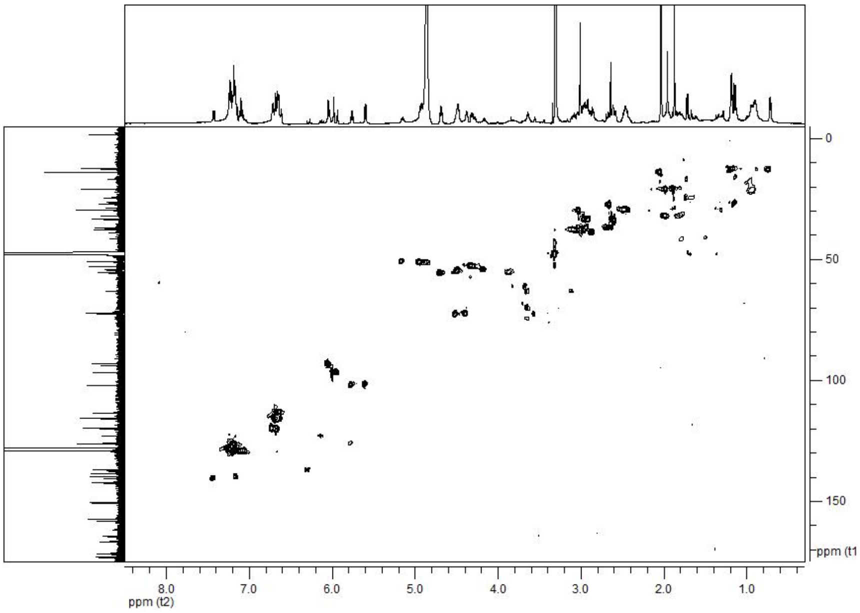


Figure S23. HSQC spectrum of component **(5)** in $CD_3OD$.

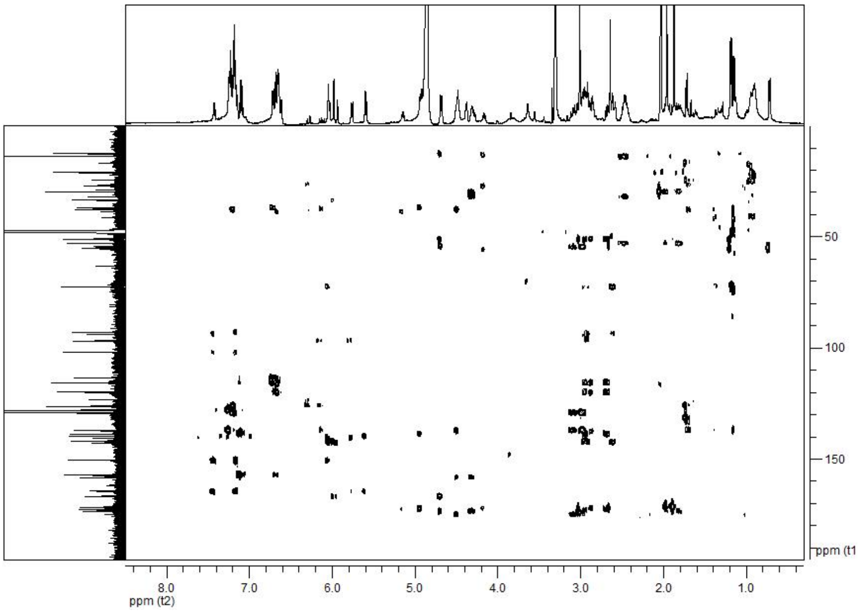


Figure S24. HMBC spectrum of component **(5)** in $CD_3OD$.

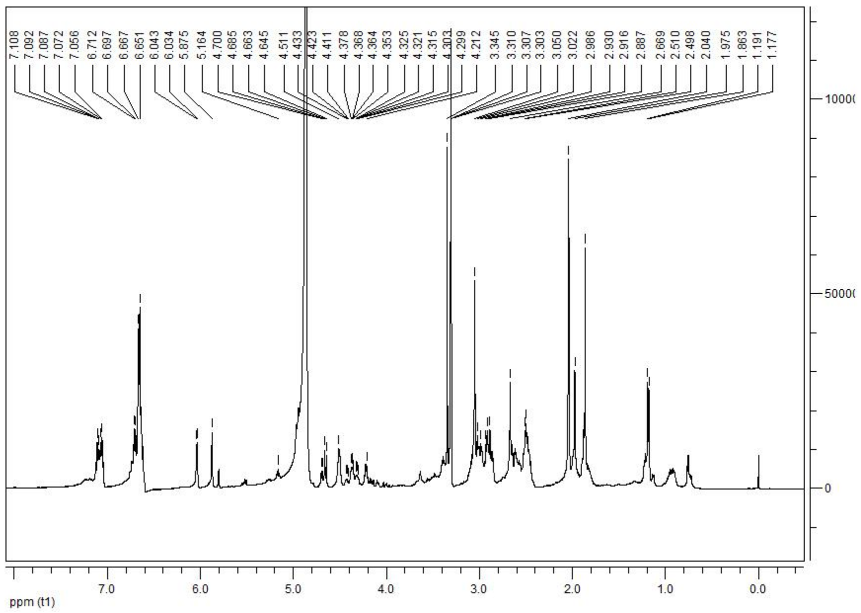

Figure S25. $^{1}H$ NMR spectrum (500 MHz) of component **(3')** in $CD_3OD$.

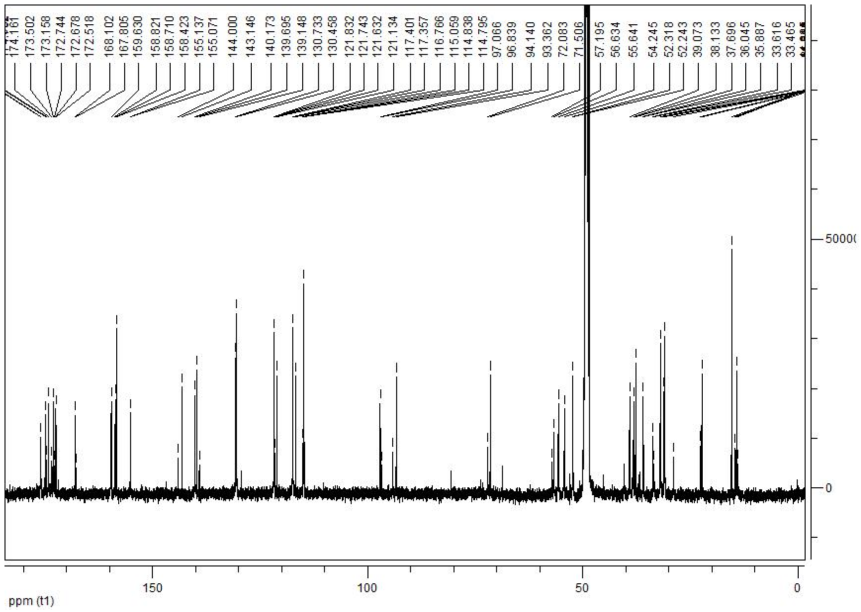

Figure S26. $^{13}C$ NMR spectrum (125 MHz) of component **(3')** in $CD_3OD$.

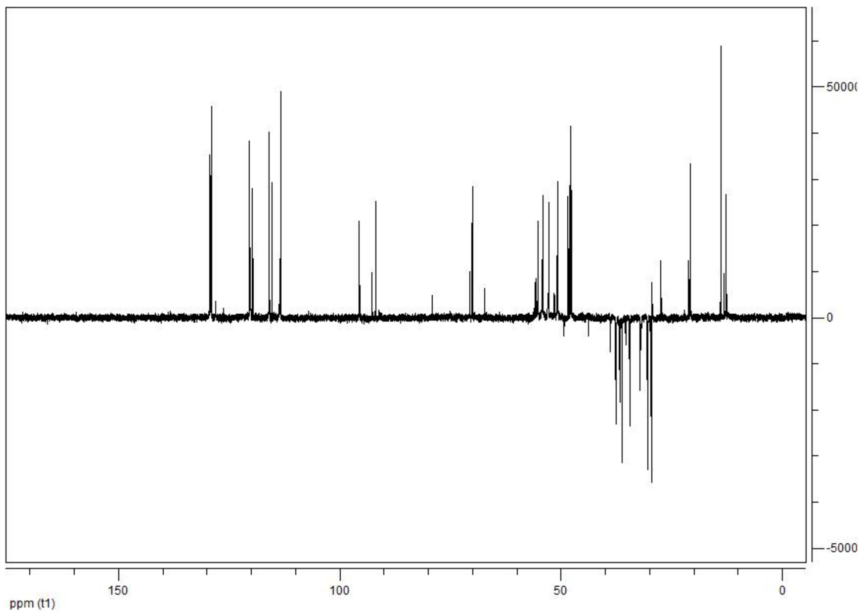


Figure S27. Dept135 spectrum of component **(3')** in $CD_3OD$.

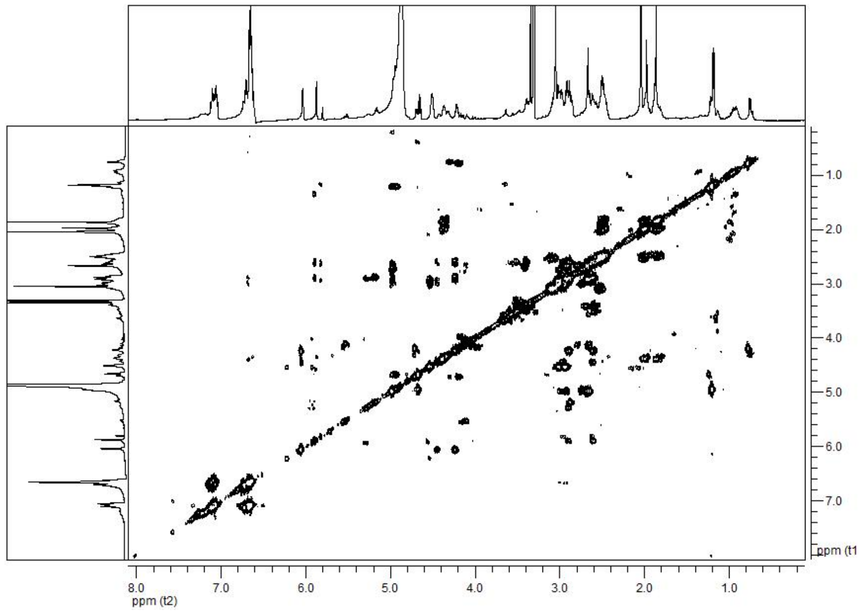


Figure S28. COSY spectrum of component **(3')** in $CD_3OD$.

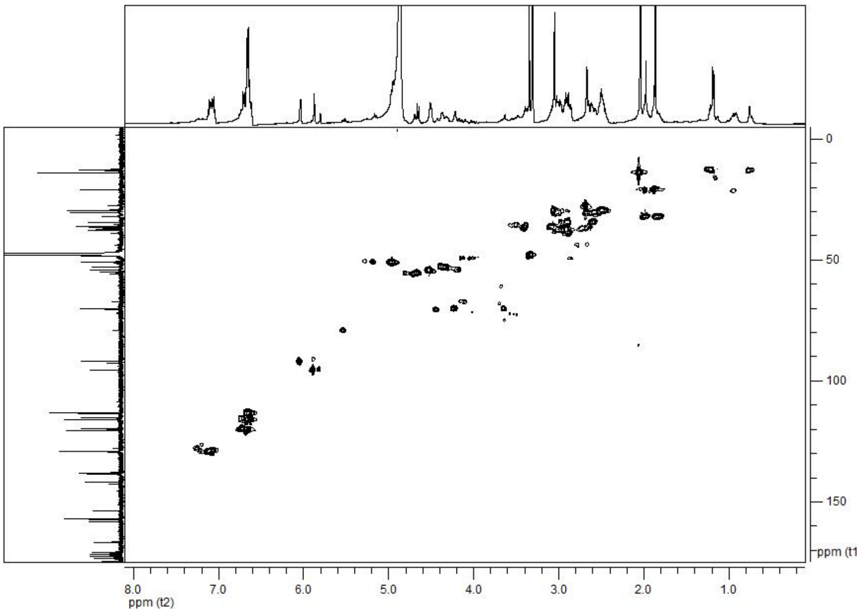


Figure S29. HSQC spectrum of component **(3')** in $CD_3OD$.

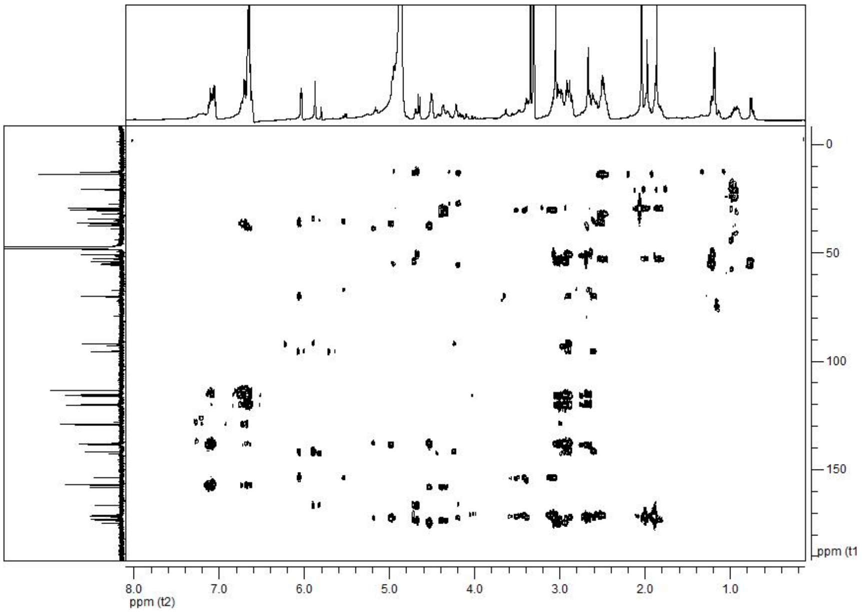


Figure S30. HMBC spectrum of component **(3')** in $CD_3OD$.

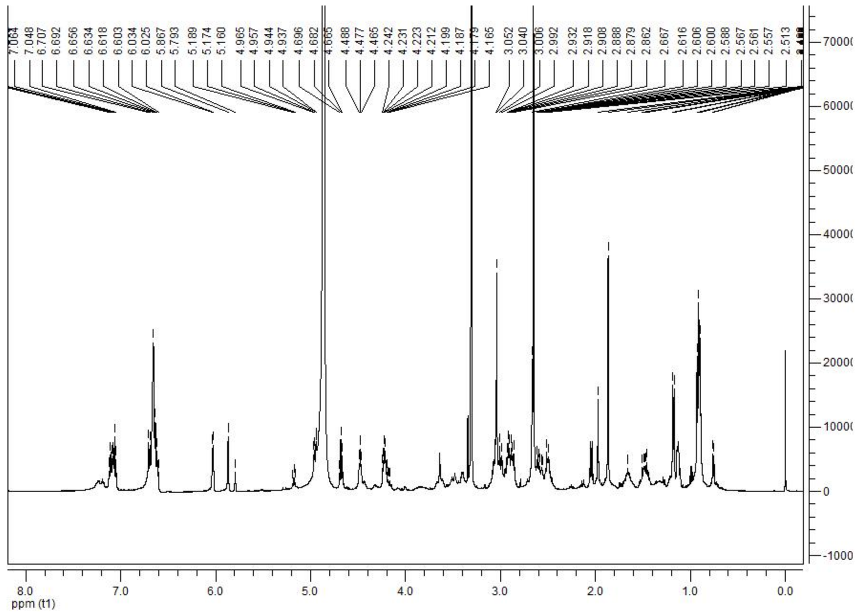

Figure S31. $^{1}$H NMR spectrum (500 MHz) of component **(4’)** in $CD_3OD$.

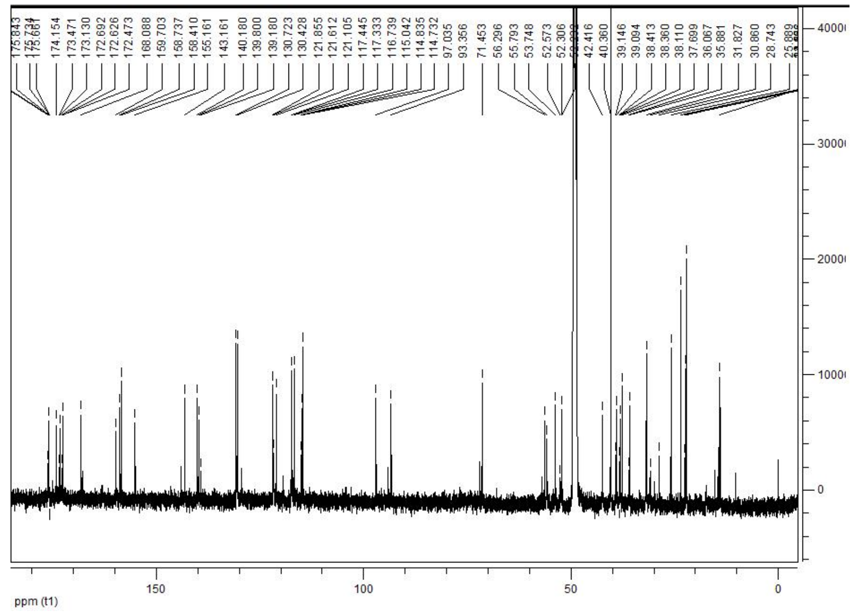

Figure S32. $^{13}$C NMR spectrum (125 MHz) of component **(4’)** in $CD_3OD$.

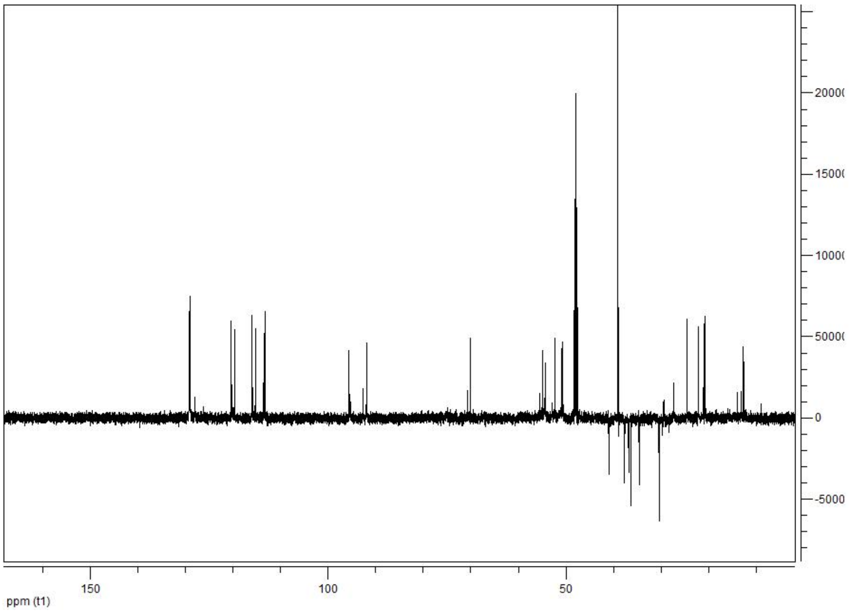


Figure S33. Dept135 spectrum of component **(4’)** in $CD_3OD$.

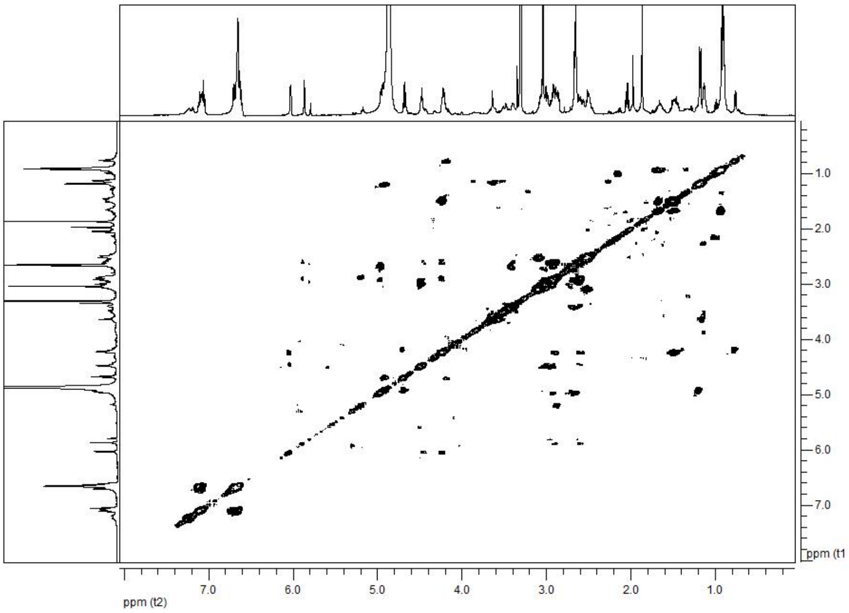


Figure S34. COSY spectrum of component **(4’)** in $CD_3OD$.

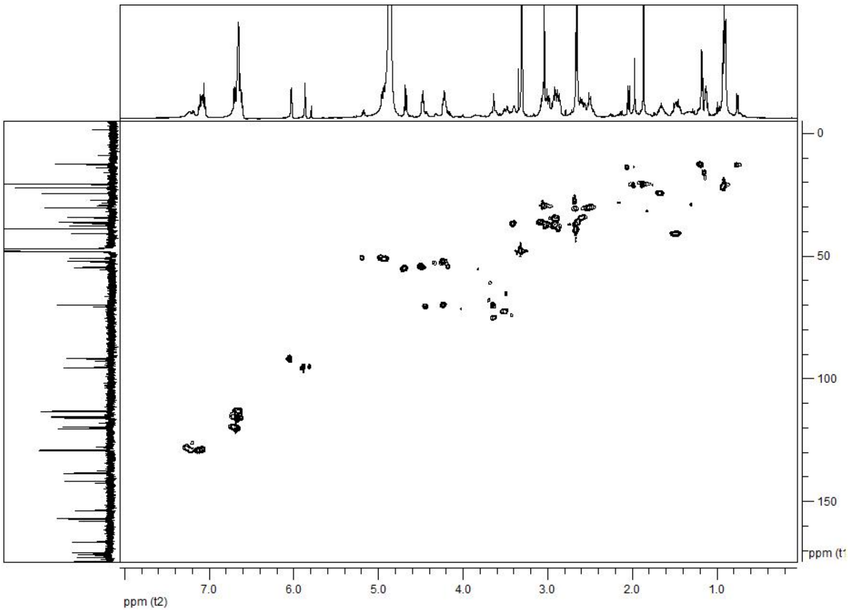


Figure S35. HSQC spectrum of component **(4')** in $CD_3OD$.

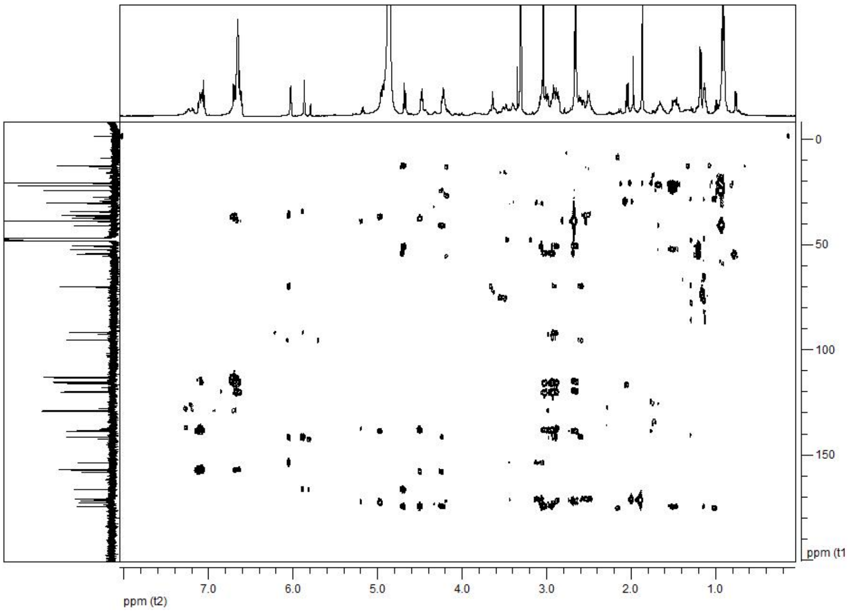


Figure S36. HMBC spectrum of component **(4')** in $CD_3OD$.

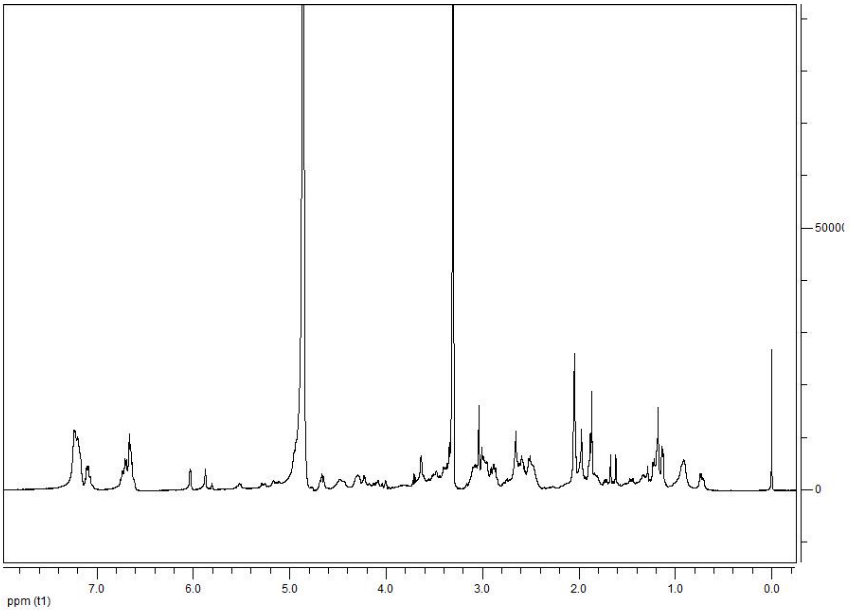


Figure S37. $^{1}$H NMR spectrum (500 MHz) of component (**5'**) in $CD_3OD$.

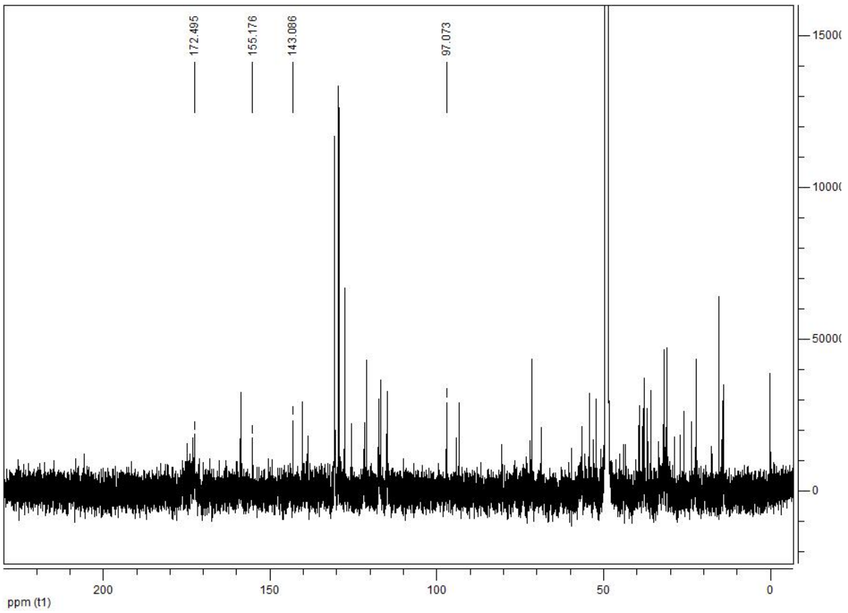


Figure S38. $^{13}$C NMR spectrum (125 MHz) of component (**5'**) in $CD_3OD$.

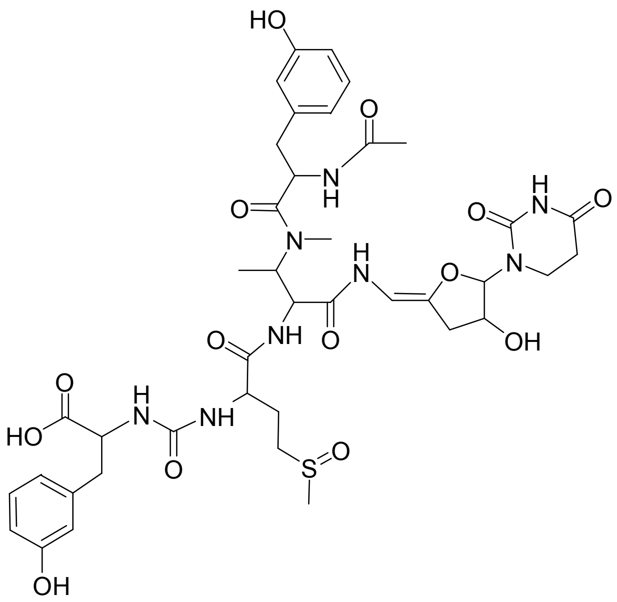


component **(2')**: *N*-acetylmureidomycin K
and *iso*-*N*-acetylmureidomycin K

Figure S39 Putative structure of compound **2'** and its isomer
