# Supplementary material for: New insights into the dihydro-mureidomycin biosynthesis controlled by two unusual proteins in Streptomyces roseosporus
Source: Microb Cell Fact. 2023 Dec 12;22:255. doi: 10.1186/s12934-023-02260-6 (PMC10714638; doi:10.1186/s12934-023-02260-6)
Supplement: Supplementary file 4 — Additional file 4. Expression of SSGG-03002 and SSGG-02980 in E. coli. Protein sequencing and modification analysis by mass spectrometry. Figure S40. Effect of SSGG-02980 disruption on the transcription of SSGG-03002. Black, Sr-hA; Red, Δ02980-hA; Blue, Δ02980c-hA. The transcription of SSGG-03002 was elevated in SSGG-02980 disruption mutant (Δ02980-hA), suggesting that SSGG-02980 could have repressive roles on the transcription of SSGG-03002; while, rather unexpectedly, the transcription in the complementary strain Δ02980c-hA did not return to the level as in starting strain Sr-hA, implying that SSGG-02980 might affect SSGG-03002 via multiple ways either directly or indirectly. So the inhibition on SSGG-03002 transcription could be just one of them. Figure S41. Expression of SSGG-03002 with or without SSGG-02980. A, Verification of the recombinant strains for overexpression of SSGG-03002 with or without SSGG-02980 in E. coli C41 (DE3). Lane 1, plasmid pET28a::02980 as reference; Lane 2, plasmids extracted from C41/03002+02980; Lane 3, the plasmid extracted from C41/03002. Lane 4, plasmid pET23b::03002 as reference. M, DNA ladder. B, SDS-PAGE analysis of the purified recombinant proteins, SSGG-03002 and SSGG-02980. Lane 1 and 2, SSGG03002 purified from strains C41/03002 and C41/03002+02980, respectively. In the latter, SSGG-02980 was co-purified with SSGG-03002 by using his-tag affinity column. M, protein standard. Figure S42. Phosphorylation of SSGG-03002 in the absence (black bar) or presence (grey bar) of SSGG-02980. The protein samples were digested following standard procedures (see Method S1 in the Additional file 4), and then subjected to LC-MS analysis for protein sequencing. The phosphorylated peptide fragments with good reproducibility in each sample were chosen for the calculation. The phosphorylation level at T304, S302 and Y305 of recombinant SSGG-03002 co-expressed with SSGG-02980 was lower than that expressed alone. However, statistic analysis indicate [file 12934_2023_2260_MOESM4_ESM.docx]

**Additional file 4**

**New insights into the dihydro-mureidomycin biosynthesis controlled by two unusual proteins in *Streptomyces roseosporus***

## Ning Liu^1^**^†^**, Yang Xu^1,2^**^†^**, Fei Shang^3^, Huiying Sun^1,2^, Xiang Liu^1,2^, Ying Huang^1^, Huarong Tan^1,2*^, Jihui Zhang^1*^

## Affiliations：

^1^ State Key Laboratory of Microbial Resources, Institute of Microbiology, Chinese Academy of Sciences, Beijing 100101, China.

^2^ College of Life Sciences, University of Chinese Academy of Sciences, Beijing 100049, China.

^3^ Analytical and Testing Center, Beijing University of Chemical Technology, Beijing 100029, China.

**Method S1**

**Expression of SSGG-03002 and SSGG-02980 in *E. coli***

To express SSGG-03002 and SSGG-02980 in *E. coli*, the ORFs of the two genes were amplified by PCR with the corresponding primer pairs 03002-F/R and 02980-F/R respectively using the genomic DNA of Sr-WT as template. Then the PCR products were inserted into *Xho*Ⅰ/*Nde*Ⅰ digested pET23b and *Eco*RI/*Hin*dIII digested pET28a via Gibson Assembly to give pET23b::*03002* and pET28a::*02980*, respectively. Finally, the plasmid pET23b::*03002* was introduced into strain C41 with or without co-transformation of pET28a::*02980.* The obtained strains were designated as C41/03002 and C41/03002+02980, then they were used for protein expression by 0.1 mM isopropyl-β-D-thiogalactopyranoside (IPTG) induction added at the exponential phase of optical density around 0.6 at OD600nm, and the induction was carried out at 16°C for 18-20 h. Then protein purification was performed using His-tag affinity column as described by the manufacture.

**Protein sequencing and modification analysis by mass spectrometry**

The proteins were digested with trypsin and subjected to an EASY-nLC 1000 interfaced via a Nanospray Flex ion source to an Orbitrap Fusion Tribrid mass spectrometer (Thermo Fisher Scientific, USA) (nano-LC-MS/MS) for analysis at the Technological Platform of Mass Spectrum Centre of Institute of Microbiology, Chinese Academy of Sciences. The peptides were loaded onto a trap column (C18, 3 μm particles, ID 100 μm , 3 cm in length, Dr. Maisch GmbH) and separated using an analytical column (C18, 1.9 μm particles, ID 150 μm , 15 cm in length, Dr. Maisch GmbH) at a flow rate of 500 nL/min with a 60 min LC gradient composed of Solvent A (0.1% formic acid) and Solvent B (acetonitrile, 0.1% formic acid). The gradient was 3-8% B for 5 min, 8-22% B for 40 min, 22-35% B for 10 min, 35-90% B for 3 min, and finally 90% B for 2 min. The mass spectrometer was operated in a data-dependent acquisition mode, in which the precursor MS1 scan (*m/z* 350–1550) was acquired in the Orbitrap at a resolution setting of 120,000, followed by Orbitrap HCD-MS/MS and OTHCD-MS/MS of the 20 most abundant multiply charged precursors in the MS1 spectrum. MS2 spectra were acquired at a resolution of 30,000.

MS/MS data were processed using Mascot search engine (v.2.8.0, 2021, http:// www.matrixscience.com; Matrix Science Ltd., London, UK). Tandem mass spectra were searched against protein sequence, and Trypsin/P was specified as cleavage enzyme allowing up to 2 missing cleavages. For precursor ions, the mass error was set to 10 ppm, and for fragment ions, the mass error was set to 0.02 Da. Carbamidomethylation on Cys was specified as fixed modification and oxidation on Met and Phosphorylation on Ser and Thr were specified as variable modifications. False discovery rate thresholds for protein, peptide and modification site were specified at 1%. All the other parameters in Mascot were set to default values.

**Figure S40. Effect of *SSGG-02980* disruption on the transcription of *SSGG-03002.***

Black, Sr-hA; Red, ∆02980-hA; Blue, ∆02980c-hA.

The transcription of *SSGG-03002* was elevated in *SSGG-02980* disruption mutant (∆02980-hA), suggesting that SSGG-02980 could have repressive roles on the transcription of *SSGG-03002*; while, rather unexpectedly, the transcription in the complementary strain ∆02980c-hA did not return to the level as in starting strain Sr-hA, implying that SSGG-02980 might affect SSGG-03002 via multiple ways either directly or indirectly. So the inhibition on *SSGG-03002* transcription could be just one of them.

**Figure S41. Expression of *SSGG-03002* with or without *SSGG-02980***

**A**,Verification of the recombinant strains for overexpression of *SSGG-03002* with or without *SSGG-02980* in *E. coli* C41 (DE3)*.* Lane 1, plasmid pET28a::*02980* as reference；Lane 2, plasmids extracted from C41/03002+02980; Lane 3, the plasmid extracted from C41/03002*.* Lane 4, plasmid pET23b::*03002* as reference*.* M, DNA ladder. **B**, SDS-PAGE analysis of the purified recombinant proteins, SSGG-03002 and SSGG-02980. Lane 1 and 2, SSGG03002 purified from strains C41/03002 and C41/03002+02980, respectively. In the latter, SSGG-02980 was co-purified with SSGG-03002 by using his-tag affinity column. M, protein standard.


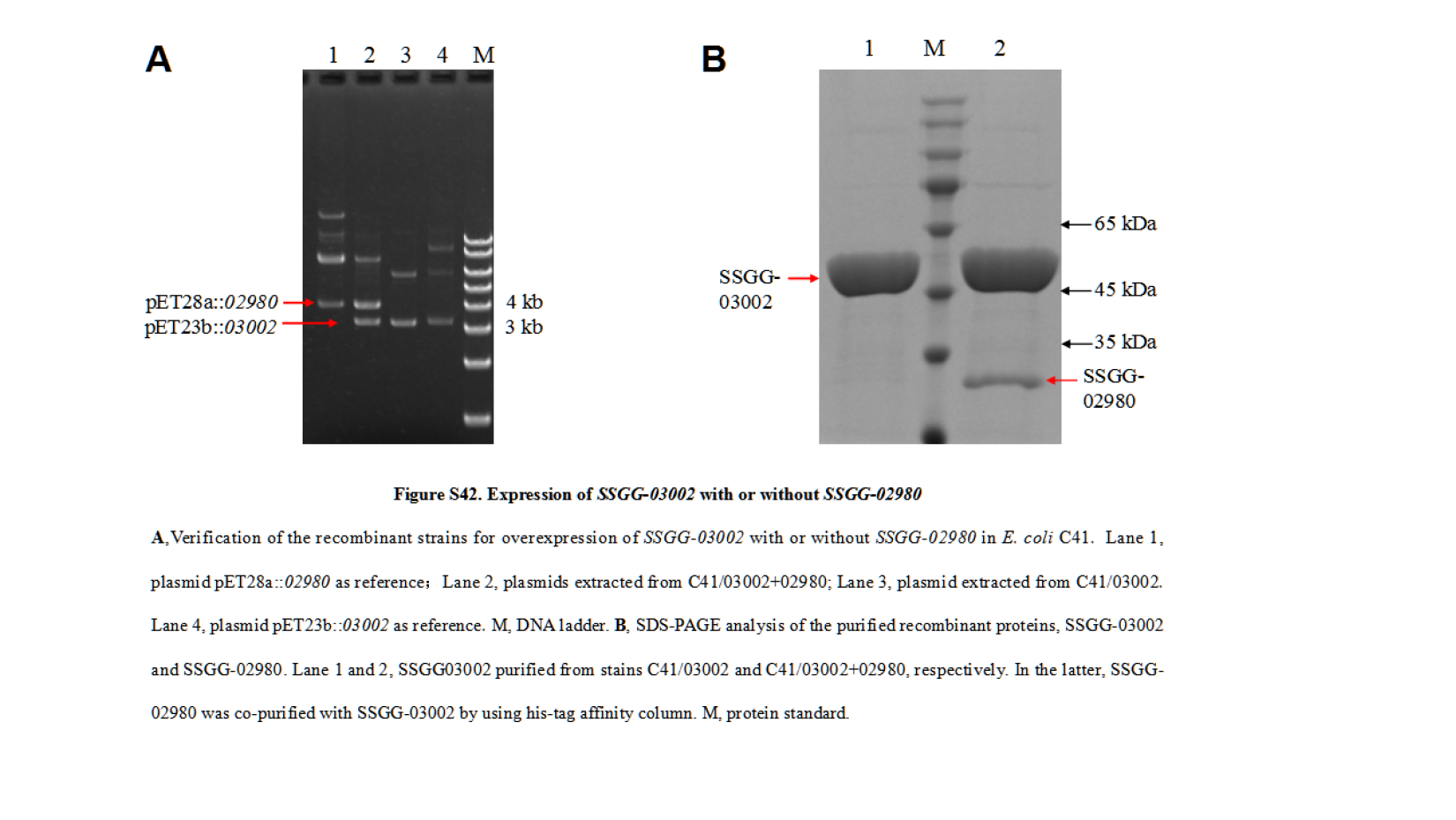


**Figure S42. Phosphorylation of SSGG-03002 in the absence (black bar) or presence (grey bar) of SSGG-02980**

The protein samples were digested following standard procedures (see Method S1 in the Additional file 4), and then subjected to LC-MS analysis for protein sequencing. The phosphorylated peptide fragments with good reproducibility in each sample were chosen for the calculation. The phosphorylation level at T304, S302 and Y305 of recombinant SSGG-03002 co-expressed with SSGG-02980 was lower than that expressed alone. However, statistic analysis indicated that the difference was not significant (*P*>0.05), so SSGG-02980 does not have considerable effect on the phosphorylation of SSGG-03002 derived from *E. coli*. Means and standard errors were calculated with the data from three independent experiments.

**
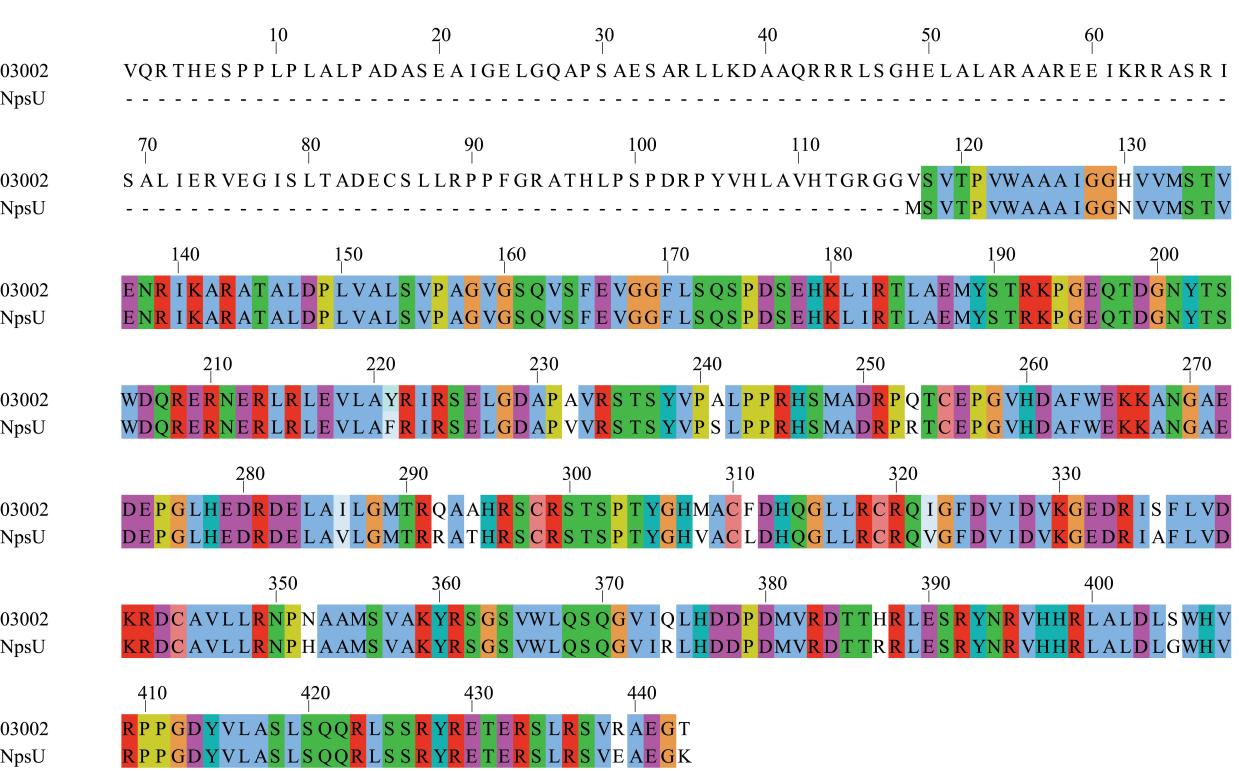
**

**Figure S43. Alignment of SSGG-03002 with NpsU**

The alignment was done with BLASTP programme, and the identity was determined to be 94.19%. The accession number of NpsU from *Streptomyces sp.* DSM 5940 in GenBank is ADY76683.1.
